# Association of Cognitive Deficit with Glutamate and Insulin Signaling in a Rat Model of Parkinson’s Disease

**DOI:** 10.3390/biomedicines11030683

**Published:** 2023-02-23

**Authors:** Ana Knezovic, Marija Piknjac, Jelena Osmanovic Barilar, Ana Babic Perhoc, Davor Virag, Jan Homolak, Melita Salkovic-Petrisic

**Affiliations:** 1 Department of Pharmacology, School of Medicine, University of Zagreb, 10000 Zagreb, Croatia; 2 Croatian Institute for Brain Research, School of Medicine, University of Zagreb, 10000 Zagreb, Croatia

**Keywords:** Parkinson’s disease, 6-hydroxydopamine, cognitive deficit, insulin, glutamate

## Abstract

Cognitive deficit is a frequent non-motor symptom in Parkinson’s disease (PD) with an unclear pathogenesis. Recent research indicates possible involvement of insulin resistance and glutamate excitotoxicity in PD development. We investigated cognitive performance and the brain glutamate and insulin signaling in a rat model of PD induced by bilateral intrastriatal injection of 6-hydroxydopamine (6-OHDA). Cognitive functions were assessed with Passive Avoidance (PA) and Morris Water Maze (MWM) tests. The expression of tyrosine hydroxylase (TH) and proteins involved in insulin (insulin receptor - IR, phosphoinositide 3 kinase - pI3K, extracellular signal-regulated kinases-ERK) and glutamate receptor (α-amino-3-hydroxy-5-methyl-4-isoxazolepropionic acid receptos-AMPAR, N-methyl-D-aspartate receptor - NMDAR) signaling was assessed in the hippocampus (HPC), hypothalamus (HPT) and striatum (S) by immunofluorescence, Western blot and enzyme-linked immunosorbent assay (ELISA). Three months after 6-OHDA treatment, cognitive deficit was accompanied by decreased AMPAR activity and TH levels (HPC, S), while levels of the proteins involved in insulin signaling remained largely unchanged. Spearman’s rank correlation revealed a strong positive correlation for pAMPAR-PA (S), pNMDAR-pI3K (HPC) and pNMDAR-IR (all regions). Additionally, a positive correlation was found for TH-ERK and TH-pI3K, and a negative one for TH-MWM/errors and pI3K-MWM/time (S). These results suggest a possible association between brain glutamate (but not insulin) signaling dysfunction and cognitive deficit in a rat PD model, detected three months after 6-OHDA treatment.

## 1. Introduction

Parkinson’s disease (PD) is the second most common age-related neurodegenerative disorder after Alzheimer’s disease (AD), with over 6 million people affected worldwide [1]. The main symptom of PD is motor deficit (bradykinesia, tremor, rigidity and gait difficulties [2]), but approximately 20–40% of PD patients suffer from cognitive impairment in the early stage, while more than 75–80% of them will eventually develop dementia, making PD the third most common form of dementia [3,4]. At the molecular level, PD is associated with degeneration of the nigrostriatal dopaminergic neurons [5] and the appearance of Lewy bodies with alpha-synuclein as the major protein associated with protein deposits [6].

Increasing evidence suggests that AD and PD share monoamine and alpha-synuclein dysfunctions, often beginning years before the onset of clinical manifestations [7]. The triggers for these impairments and the causes leading these early neurodegenerative processes to develop further in the form characteristic of AD or PD remain unclear [7]. Currently, the background of cognitive dysfunction in PD is not fully understood and is still a matter of an ongoing debate [8]. Latest research indicates a possible connection between insulin resistance in the brain and the development of dementia in PD [9] and suggests that metabolic dysfunction might be an important player in the pathophysiology of PD [10,11,12,13,14]. Moreover, recent research indicates a possible link between type 2 diabetes (T2DM) and PD: (a) they may have a common pathological mechanism and (b) presence of T2DM increases the risk for PD [15].

As with AD, there is a possibility that glutamate excitotoxicity is also involved in the development of PD [16]. Previous studies have shown that glutamate excitotoxicity may induce degeneration of the dopaminergic neurons and concomitant motor dysfunction in PD [17,18]. Several clinical results have revealed slight alterations in glutamate content in the brain of PD patients post mortem that indicate increased glutamate neurotransmission [19]. The excitotoxic glutamate cascade in PD is triggered by excess extracellular glutamate, which can ultimately lead to cell injury and death. With the excessive activation of N-methyl-D-aspartate (NMDA) receptors, the increased influx of Ca^2+^ ions worsens further the level of reactive oxygen compounds, leads to mitochondrial damage and increases susceptibility to cell death. In addition, excessive activation of α-amino-3-hydroxy-5-methyl-4-isoxazolepropionic acid (AMPA) receptors causes Na^+^ ion overload, resulting in high intracellular permeability and acute cell swelling [19]. Data concerning the interconnection of the glutamate and insulin signaling, in regards to cognitive and motoric deficit, and in relation to the affected brain regions, are missing. Therefore, to further explore this aspect of PD pathophysiology, it is necessary to use animal models.

There are two leading approaches to non-transgenic models of PD generated by exogenous toxins; administration of 1-methyl-4-phenyl-1,2,3,6-tetrahydropyridine (MPTP) or 6-hydroxydopamine (6-OHDA) [20]. The most commonly used rat model in preclinical research of PD is based on the central application of 6-OHDA, directly into the target brain region. In the brain, 6-OHDA has the ability to induce degeneration of dopaminergic and noradrenergic neurons. These types of neurons are particularly sensitive to 6-OHDA because their membrane transport proteins have a high affinity for this molecule [21]. Once absorbed, 6-OHDA accumulates in the neuronal cytosol, where it leads to progressive neuronal deterioration due to oxidative stress-induced cytotoxicity [22,23]. The usual locations of 6-OHDA administration are the substantia nigra pars compacta (SNpc), the medial forebrain bundle (MFB) or the striatum [24]. Unilateral administration of 6-OHDA into the SNpc or MFB causes a quick and vast degeneration of dopaminergic cell bodies (and anterograde progression of neurodegeneration), while injection into the striatum primarily affects the dopaminergic terminals (with further retrograde progression of degeneration) [25]. Intrastriatal administration of 6-OHDA causes a full array of premotor Parkinsonian symptoms, including cognitive deficit, with incomplete nigrostriatal cell loss and partial striatal dopamine depletion similar to early stages of the disease, and gradual SN cell loss, thus representing PD more closely [25,26,27,28].

The aim of this research was to clarify the connection between degeneration of dopaminergic neurons, motor and cognitive deficits with glutamate and insulin signaling in relation to the brain regions involved in cognition (hippocampus), motor function (striatum) and metabolism (hypothalamus) in an animal PD model induced by bilateral intrastriatal 6-OHDA application.

## 2. Materials and Methods

### 2.1. Animals

The research was conducted on adult, 3-month-old (equivalent to young adults [29]), male *Wistar* rats, (University of Zagreb, School of Medicine, Department of Pharmacology). All animals were housed (2–3 rats per cage) from 1 month of age and during the whole experiment (in total 5 months) in a licensed animal facility at the Department, kept on standardized food pellets and tap water ad libitum and maintained under a 12/12 h light/dark cycle.

### 2.2. Ethics

All procedures involving animals, their care, administration of drugs, in vivo tests and sacrifice were carried out in accordance with institutional guidelines that comply with national and international laws: (1) Directive of the European Parliament and the Council for the Protection of Animals Used for Scientific Purposes; (2) Croatian Law on Animal Welfare (NN 135/06); (3) Act on Changes and Amendments to the Croatian Animal Welfare Act (NN 37/13); (4) Croatian Law on Animal Protection (OG 102/17); (5) Guidelines for the care of animals in laboratory procedures or for other scientific purposes (NN55/13); (6) Guidelines for laboratory animal anesthesia procedures. In vivo experiments on animals, cognitive testing and animal sacrifice were approved by the Ethics Committee/Committee for Animal Welfare of the School of Medicine, University of Zagreb, as well as by the Ministry of Agriculture (CLASS: UP/1-322-01/18-01/57; NUMBER: 525-10/0255-18-5). The animals were bred and housed in a registered animal facility at the Department of Pharmacology, School of Medicine, University of Zagreb. Procedures performed on animals were carried out in compliance with the 3R principle. The persons who handled the animals were officially trained to work with laboratory animals.

### 2.3. Experimental Rat Model of Parkinson’s Disease

Under general anesthesia (ketamine 70 mg/kg/xylazine 7 mg/kg ip), rats were administered with 6-OHDA bilaterally into the striatum (caudate putamen, coordinates: AP-0 mm; ML-3 mm; DV-7 mm) in a total dose of 16 µg (dissolved in 0.02% ascorbic acid; 8 µg in 2 µL per brain hemisphere; OHDA; *n* = 14) [21,30]. An equal volume of solvent (0.02% ascorbic acid) was applied intrastriatally to control animals using the same procedure (CTRis; *n =* 14) and a third group of animals was not treated (intact group; CTRint; *n =* 9).

### 2.4. Cognitive Testing

Three months after 6-OHDA administration, the animals were subjected to cognitive and motor tests. Cognitive functions of learning and memory were tested with two standard tests: the Morris Water Maze Swimming Test (MWM) and the Passive Avoidance Test (PA) and the motor ability of the animals was tested using the rotating cylinder test (results on the motor ability published in preprint [31], currently under review).

### 2.5. MWM

The Morris Water Maze Swimming Test (MWM) was conducted [32] in a 180 cm diameter pool with water at a standard temperature of 25 °C. The testing itself was performed using the Ethovision XT animal monitoring software (Version 11.5, Noldus, Wageningen, the Netherlands), with a cut-off time of 1 min. The pool was divided into 4 equal quadrants: NW—northwest, NE—northeast, SW—southwest, SE—southeast. A hidden underwater platform was located in the NW quadrant. Testing was conducted over 6 consecutive days. The time required to find the platform over 5 consecutive days (4 tests per day) and the number of entries (errors) into quadrants without the platform (learning phase) were measured. On the last day of testing, the platform was removed and the time spent in search of the platform in the correct quadrant and the number of entries/errors into other compartments were recorded in the probe trial.

### 2.6. PA

The Passive Avoidance Test (PA) apparatus (Ugo Basile, Gemonio, Italy) is divided into 2 compartments, a light and a dark one, with a sliding door between them. The time required for the animal to enter the dark compartment was measured one day after the animal was subjected to an electric shock of 0.5 mA for 2 s. On the first day, habituation to the new environment was carried out—the animals explored both compartments without being subjected to any electric shock, and after entering the dark compartment, they were taken out of the apparatus after 15 s. The next day, the animals were subjected to an electric shock after entering the dark compartment, in order to learn to avoid the dark chamber. On the third day, the time required for the animal to enter the dark compartment was measured with a cut-off time of 5 min [33].

### 2.7. Tissue Preparation

After cognitive testing, the animals were sacrificed under deep anesthesia (thiopental 70 mg/kg/diazepam 7 mg/kg). The animals were decapitated and their brain removed. The hippocampus (HPC), hypothalamus (HPT) and striatum (S) were isolated from half of the brain, for protein analysis by Western blotting (WB) and enzyme-linked immunosorbent assay (ELISA) analysis. The samples were stored at −80 °C. For protein isolation and subsequent measurement of protein levels, HPC, HPT and S were homogenized using an ultrasonic homogenizer (Microson Ultrasonic Cell Disruptor XL; Misonix, Farmingdale, NY, USA) in cell lysis buffer solution (1 M Tris (tris(hydroxymethyl)aminomethane) pH 8.0; 1 M NaCl; 0.005 M EDTA; 1 M DTT; 0.01 M sodium vanadate; 1% NP-40) with protease (Roche Holding AG, Basel, Switzerland; Cat.#04693132001) and phosphatase (Roche Holding AG, Basel, Switzerland; Cat.#4906837001) inhibitors. Homogenates were centrifuged for 10 min at 12,500 rpm at 4 °C (Biofuge Fresco Heraeus, Hanau, Germany), and supernatants were stored at −80 °C [34]. The protein concentration was measured using the Lowry protein assay [35]. The remaining brain halves were washed in cold saline and immersed in 4% buffered paraformaldehyde (PFA) for 2 days. After fixation, animal brains were cryopreserved with serial sucrose solutions (15% and 30%). Then, the brain was embedded in Tissue-Tek solution using Tissue-Tek Cryomolds (Sakura Finetek USA, Torrance, CA, USA). Brains were sliced using a cryostat (Leica CM1850; Wetzlar, Germany) (16 μm), mounted on slides and dried overnight at 37 °C before the immunofluorescence procedure.

### 2.8. Immunofluorescence

Previously sliced and dried sections were washed 3 times for 5 min in phosphate buffer (PBS). Non-specific binding sites were blocked with 10% normal goat serum (NGS) in PBST (0.25% Triton X-100 in PBS) for 1 h at room temperature and incubated overnight at 4 °C with the primary anti-tyrosine hydroxylase (TH) antibody (Merck Millipore, Burlington, MA, USA; Cat.#AB152) diluted in 1% NGS in PBST at a concentration of 1:1000. After the incubation, slides were washed in PBS (3 × 5 min) and incubated with the appropriate secondary fluorescent antibody (1:500) for 2 h in the dark (anti-rabbit Alexa Fluor 555; Cell Signaling Technology, Danvers, MA, USA; Cat.#4413). After incubation, the sections were washed and slides were again incubated with the primary antibody NeuN (1:1000) (Merck Millipore, Burlington, MA, USA; Cat.#MAB377) and the next day with the appropriate secondary fluorescent antibody (anti-mouse Alexa Fluor 488; 1:600; Cell Signaling Technology, Danvers, MA, USA; Cat.#4408). The sections were then washed again in PBS 3 times, allowed to dry for a short period and coverslipped with Fluoroshield mounting medium with DAPI (Sigma-Aldrich, St. Louis, MI, USA; Cat.#F6057). Slides were viewed and analyzed using the Olympus BX51 microscope and the CellSense Dimension software (Version 1.6, Olympus Corporation, Shinjuku City, Tokio, Japan) [36].

### 2.9. Insulin Concentration Measurement

Insulin concentration in the brain was determined by commercially available enzyme immunoassay (Rat/Mouse Insulin ELISA, Merck Millipore, Burlington, MA, USA, Cat.#EZRMI-13K) in strict compliance with the manufacturer’s protocol and analyzed by colorimetric analysis using a multimodal microplate reader Infinite F200 PRO (Tecan, Männedorf, Switzerland). Rat brain samples were added to the corresponding wells for insulin measurement. Absorbance was measured at 450 nm, subtracted for absorbance at 590 nm using a microplate reader. The concentration of insulin was calculated using the absorbance curve of the standard and was expressed in ng/mL.

### 2.10. Western Blot Analysis

Samples for electrophoresis were prepared by mixing equal amounts of homogenate and buffer (2 mL glycerol; 6 mL 10% SDS; 2.5 mL 1 M tris buffer pH 6.7; 2–4 mg bromophenol blue) and 10% β-mercaptoethanol was added. Samples were briefly heated at 95 °C for 5 min and centrifuged afterwards for 60 s at 13,000 rpm (Mikro 120; Hettich, Westphalia, Germany). Equal amounts of protein (40 µg for HPC and 30 µg for HPT and S) were separated by sodium dodecyl sulphate–polyacrylamide gel electrophoresis (SDS-PAGE) using 12% TGX Stain-Free gels (TGX Stain-Free FastCast acrylamide kit; Bio-Rad Laboratories, Hercules, CA, USA; Cat.#1610185). After electrophoresis, gels were visualized using the ChemiDoc Imaging System (Bio-Rad Laboratories, Hercules, CA, USA) and transfer efficiency was checked after transfer using the same technology. All the proteins in the sample were used for normalization and analysis. Following visualization, proteins were transferred to nitrocellulose membranes which were then blocked for 1 h at RT in 5% non-fat milk in a low-salt washing buffer (LSWB; 10 mM Tris, 150 mM NaCl, pH 7.5 and 0.5% Tween 20). After blocking, the membranes were incubated overnight at 4 °C with primary antibodies to detect the enzyme involved in dopamine synthesis (anti-TH), insulin receptor (IR, anti-IR; Merck Millipore, Burlington, MA, USA; Cat.#07-724) as well as two types of glutamate receptors (anti-total-AMPAR/tAMPAR/, Cat.#AB1504, anti-phospho-AMPAR/pAMPAR/, Cat.#AB5849, anti-phospho-NMDAR/pNMDAR, Cat.#ABN99; Merck Millipore, Burlington, MA, USA) and IR-signaling downstream elements (anti-phosphoinositide 3 kinase p85 (PI3K, Cat.#4257), anti-extracellular signal-regulated kinases (ERK, Cat.#4695; Cell Signaling Technology, Danvers, MA, USA; 1:1000 dilutions). After incubation, membranes were washed 3x with LSWB and incubated for 1 h at RT with the appropriate secondary antibody solution (anti-rabbit or anti-mouse, 1:2000; Cell Signaling Technology, Danvers, MA, USA), washed in LSWB and incubated with chemiluminescent reagent (Super Signal West Femto; Thermo Fisher Scientific, Waltham, MA, USA; Cat.#34095). The signals were captured and visualized with a MicroChemi video camera system (DNR Bio-Imaging Systems MicroChemi, Jerusalem, Israel). The ImageJ software (ImageJ; U.S. National Institutes of Health, Bethesda, MD, USA) was used to analyze the bands obtained by the Western blotting method. Total proteins were used as a control of the procedure, and protein intensities were expressed in proportion to the signal of total proteins in the same sample on the corresponding membrane [34].

### 2.11. Statistics

Protein levels and PA test results were presented as box and whiskers (min to max) plots with between-group differences tested by two-tailed Kruskal–Wallis one-way analysis of variance, followed by the Mann–Whitney U-test, with *p* < 0.05 considered statistically significant, using the GraphPad Prism 5 statistical software. MWM test results for learning trials were expressed as mean ± SD and analyzed by two-way ANOVA for repeated-measures with Bonferroni post-hoc correction, with *p* < 0.05 considered statistically significant, using the GraphPad Prism 5 statistical software. Principal component analysis was used for multivariate exploratory data analysis following data normalization using the Jamovi software (Version 2.3, Sidney, Australia). The results were presented as coordinates of individuals in respect to the biplot, and vectors of variables indicating contributions to the first two principal components (PC). Monotonic associations were analyzed by calculating Spearman’s rank correlation coefficients (ρ) for pairs of measured variables. Correlations were reported as heatmaps visualized in the JASP software (Version 0.15, Amsterdam, The Netherlands).

## 3. Results

### 3.1. 6-OHDA Administration Induced Learning and Memory Deficit

**Figure 1:**
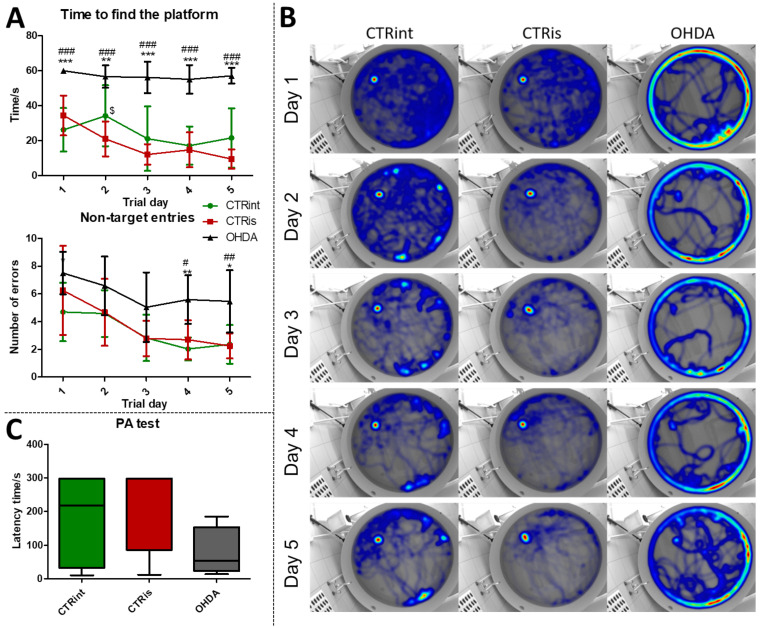
The 6-OHDA induces learning and memory deficit. Learning and memory functions were tested with the Morris maze swimming test (MWM) and Passive Avoidance test (PA) 3 months after intrastriatal administration of 6-OHDA (OHDA). (**A**) Each dot represents a mean value ± SD of the time spent in search for the platform or number of entries into quadrants without platform (non-target entries) during 5 consecutive days. Group comparisons were analyzed by two-way ANOVA for repeated-measures with Bonferroni post-hoc test. (**B**) Merged heatmaps of groups of animals in MWM during 5 consecutive trial days. The color of a pixel represents the total time an animal was at a certain location and the heatmap is based on the all animals’ locations. (**C**) Results (latency time) of the fear-conditioned learning PA test are represented as a box and whiskers (min to max) plot with between-group differences tested by two-tailed Kruskal–Wallis one-way analysis of variance, followed by the Mann–Whitney U-test, with *p* < 0.05 considered statistically significant (* *p* < 0.05, ** *p* < 0.01, *** *p* < 0.001 vs. CTRint; # *p* < 0.05, ## *p* < 0.01, ### *p* < 0.001 vs. CTRis; $ *p* < 0.05 CTRint vs. CTRis). *n* (intact control/CTR) = 9, *n* (sham operated control/CTRis) = 13, *n* (OHDA) = 6.

In the MWM test, animals in the OHDA group spent a longer time to find the platform during the learning trials (Figure 1A), compared to both controls (CTRint and CTRis; +65.4%–+503.6%; *p* < 0.001). In addition, animals in the OHDA group entered the wrong quadrants (non-target entries) more often (Figure 1A), in comparison to both controls during the fourth (+107.4% vs. CTRis; *p* < 0.05, +175.3% vs. CTRint; *p* < 0.01) and fifth (+131.2% vs. CTRint; *p* < 0.05, +142.8% vs. CTRis; *p* < 0.01) day of the learning trial. Cumulative trajectories of animals separated by treatment depicted in heatmaps (Figure 1B) point to one more observation between control animals and OHDA-treated ones: the tendency of OHDA animals to stay in the proximity of the MWM pool walls, indicating possible anxious behavior.

Although there is no statistically significant difference in the latency time between the groups in the PA test, there is a tendency of OHDA animals to spend less time in the light compartment compared to controls (Figure 1C). In the probe trial, OHDA rats spent less time in search of the platform compared to both control groups: CTRint (−69.9%, *p* = 0.0221) and CTRis (−61.2%, *p* = 0.0352) (Supplement S1 (Supplementary Materials)) and had less non-target entries compared to CTRis (−47%, *p* = 0.0096) (Supplement S1).

In addition, and as expected, the 6-OHDA model was characterized by a relatively large dropout rate throughout the experiment while no fatal outcomes were observed in either of the two control groups. Rotarod performance showed pronounced motor deficits in the 6-OHDA group. The results of motor findings are presented in the previously published manuscript [31].

### 3.2. 6-OHDA Administration Induced Dopaminergic Neurodegeneration (Reduced TH Levels) in the Cortex, HPC, HPT and S, while Dopaminergic Nuclei in the Substantia Nigra Were Found Preserved

**Figure 2:**
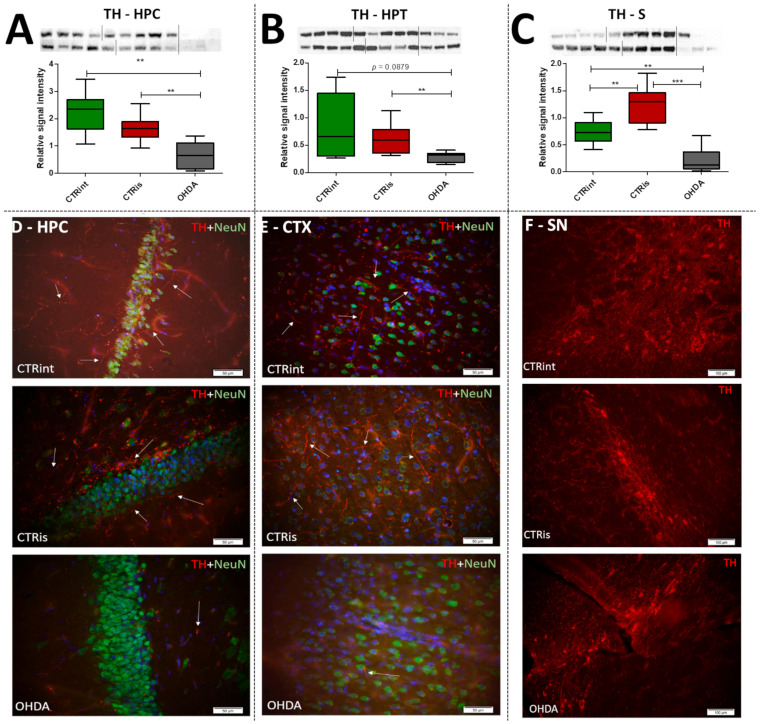
The effect of intrastriatal administration of 6-OHDA on brain tyrosine hydroxylase levels. Protein levels of tyrosine hydroxylase (TH) were measured by Western blot (**A**–**C**) and immunofluorescence (**D**–**F**) in the brain of rats 3 months after intrastriatal administration of 6-OHDA (OHDA) or vehicle only (CTRis) and aged matched intact rats (CTRint). The Western blot results of TH levels in the hippocampus (HPC, (**A**)), hypothalamus (HPT, (**B**)) and striatum (S, (**C**)) are represented as a box and whiskers (min to max) plot with between-group differences tested by two-tailed Kruskal–Wallis one-way analysis of variance, followed by the Mann–Whitney U-test, with *p* < 0.05 considered statistically significant (** *p* < 0.01, *** *p* < 0.001). Corresponding Western blot images are presented above the plot. Images of immunofluorescence show TH positive signals(red staining), a neuronal marker (NeuN; green staining) and DAPI (blue staining) in representative sections of the parietal cortex (CTX, (**E**); scale bar—50 µm), hippocampus (HPC, (**D**); scale bar—50 µm) and substantia nigra (SN, (**F**); scale bar—100 µm) in CTRint, CTRis and OHDA animals. Images were captured and merged using the cellSens Dimension software. The white arrows indicate a positive TH signal in HPC and CTX. *n* (intact control/CTR) = 9, *n* (sham operated control/CTRis) = 9, *n* (OHDA) = 6.

Levels of TH determined by Western blot were found decreased in all observed regions (Figure 2A–C) compared to CTRis (in HPC −59.8%, *p* = 0.0028; in HPT −52.7%, *p* = 0.0076; in S −82.1%, *p* = 0.0004) and compared to the intact controls (in HPC −70.4%, *p* = 0.0016; in S −70.9 %, *p* = 0.0048) except in the HPT, where there was significant data dispersion in the CTRint group. Three months after intrastriatal vehicle administration (CTRis), striatal TH levels were found increased in comparison to intact controls (Figure 2C) (+68.8%, *p* = 0.0040). Immunofluorescent analysis confirmed decrement of TH positive signal (red) in the cortex (Figure 2E) and hippocampus (Figure 2D) compared to both controls. Contrary to the observed decrease in TH levels, intrastriatal administration of 6-OHDA did not induce the loss of TH positive cells in the substantia nigra, compared to both control groups (Figure 2F).

### 3.3. A Total of 3 Months after 6-OHDA Administration, No Changes Were Observed in the Level of Proteins Involved in Insulin Signaling

**Figure 3:**
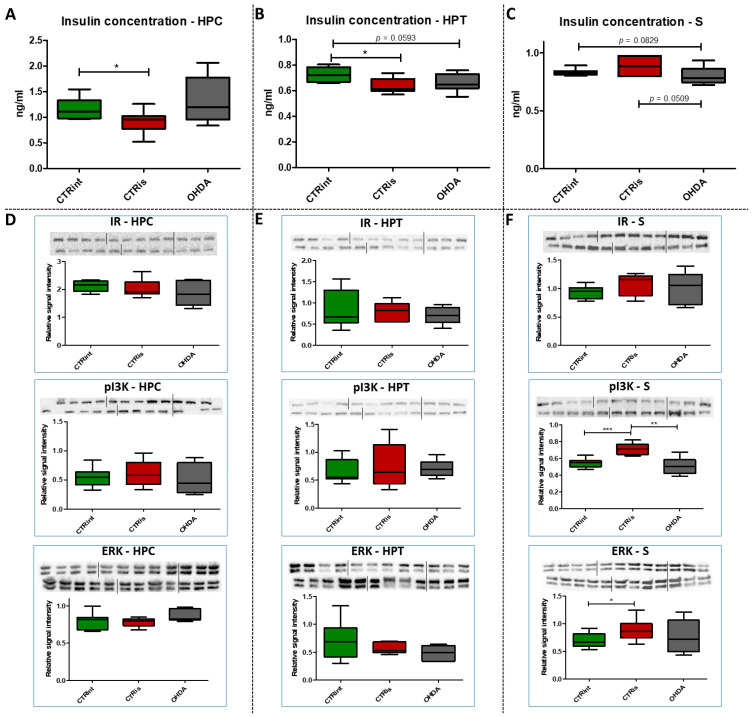
The effect of intrastriatal administration of 6-OHDA on protein levels involved in insulin signaling. Insulin concentration was measured by ELISA (**A**–**C**) and protein levels of insulin receptor (IR), phosphoinositide 3-kinases (pI3K) and extracellular signal-regulated kinase (ERK) were measured by Western blot (**D**–**F**) in the brain of animals 3 months after intrastriatal administration of 6-OHDA (OHDA) or vehicle only (CTRis) and aged matched intact rats (CTRint). ELISA and Western blot results in the hippocampus (HPC, (**A**,**D**)), hypothalamus (HPT, (**B**,**E**)) and striatum (S, (**C**,**F**)) are represented as a box and whiskers (min to max) plot with between-group differences tested by two-tailed Kruskal–Wallis one-way analysis of variance, followed by the Mann–Whitney U-test, with *p* < 0.05 considered statistically significant (* *p* < 0.05, ** *p* < 0.01, *** *p* < 0.001). Corresponding Western blot images are presented above the plot. *n* (intact control/CTR) = 9, *n* (sham operated control/CTRis) = 9, *n* (OHDA) = 6.

The administration of 6-OHDA did not induce statistically significant changes in insulin concentration measured by ELISA compared to intact rats (CTRint) and rats that received vehicle only (CTRis) in all observed regions (Figure 3A–C). However, a tendency towards a decrease in insulin concentration after intrastriatal administration of OHDA was noticed in HPT (−9% vs. CTRint, *p* = 0.0593; Figure 3B) and S (−4% vs. CTRint, *p* = 0.0829; −9% vs. CTRis, *p* = 0.0509; Figure 3C). Statistically significant decrement of insulin concentration was observed after intrastriatal administration of vehicle only compared to intact controls in the HPC (−21.8%, *p* = 0.0244; Figure 3A) and HPT (−11.5%, *p* = 0.0206; Figure 3B). The analysis of protein levels involved in insulin signaling (IR, pI3K, ERK) did not show statistically significant changes in the HPC (Figure 3D) and HPT (Figure 3E). Intrastriatal administration of vehicle only induced increase in pI3K (+31.5% vs. CTRint, *p* < 0.0001; +39.9% vs. OHDA, *p* = 0.0028) and ERK (+18.6% vs. CTRint, *p* = 0.0315) levels in the striatum (Figure 3F).

### 3.4. 6-OHDA Induced Decrement of Phosphorylated AMPAR Levels in the HPC and S

**Figure 4:**
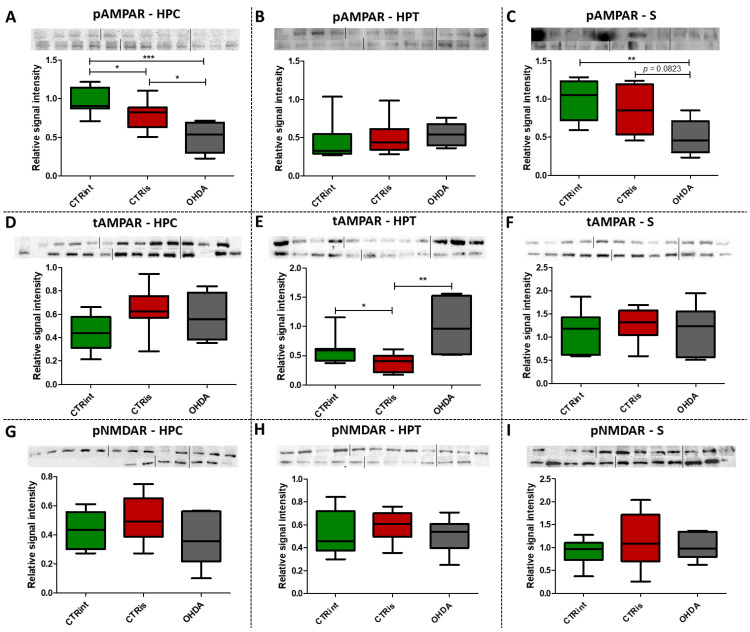
The effect of intrastriatal administration of 6-OHDA on levels of glutamate receptors. Protein levels of phosphorylated (pAMPAR; (**A**–**C**)) and total AMPAR (tAMPAR; (**D**–**F**)) receptors and levels of phosphorylated NMDAR receptors (pNMDAR; (**G**–**I**)) were measured by Western blot in the brains of animals 3 months after intrastriatal administration of 6-OHDA (OHDA) or vehicle only (CTRis) and aged matched intact rats (CTRint). Western blot results in the hippocampus (HPC; A, (**D**,**G**)), hypothalamus (HPT; (**B**,**E**,**H**)) and striatum (S; (**C**,**F**,**I**)) are represented as a box and whiskers (min to max) plot with between-group differences tested by two-tailed Kruskal–Wallis one-way analysis of variance, followed by the Mann–Whitney U-test, with *p* < 0.05 considered statistically significant (* *p* < 0.05, ** *p* < 0.01, *** *p* < 0.001). Corresponding Western blot images are presented above the plot. *n* (intact control/CTR) = 9, *n* (sham operated control/CTRis) = 9, *n* (OHDA) = 6.

Western blot analysis showed a statistically significant decrease in pAMPAR levels 3 months after 6-OHDA treatment compared to controls in the HPC (−49.2% vs. CTRint, *p* = 0.0008; −36.3% vs. CTRis, *p* = 0.0176; Figure 4A) and S (−49.9% vs. CTRint, *p* = 0.0047; −42.4% vs. CTRis, *p* = 0.0823; Figure 4C). Intrastriatal administration of vehicle only induced decreases in the pAMPAR levels in the HPC (−20.3%, *p* = 0.0188; Figure 4A) and tAMPAR levels in the HPT (−37.5%, *p* = 0.0464; Figure 4E) compared to the intact control group, while tAMPAR levels were found increased in the OHDA group compared to CTRis in the HPT (+166.3%, *p* = 0.0048; Figure 4E). There was no statistically significant change of pNMDAR levels in all of the observed regions 3 months after the 6-OHDA injection (Figure 4G–I).

### 3.5. 6-OHDA Induces Regionally Specific Correlations

**Figure 5:**
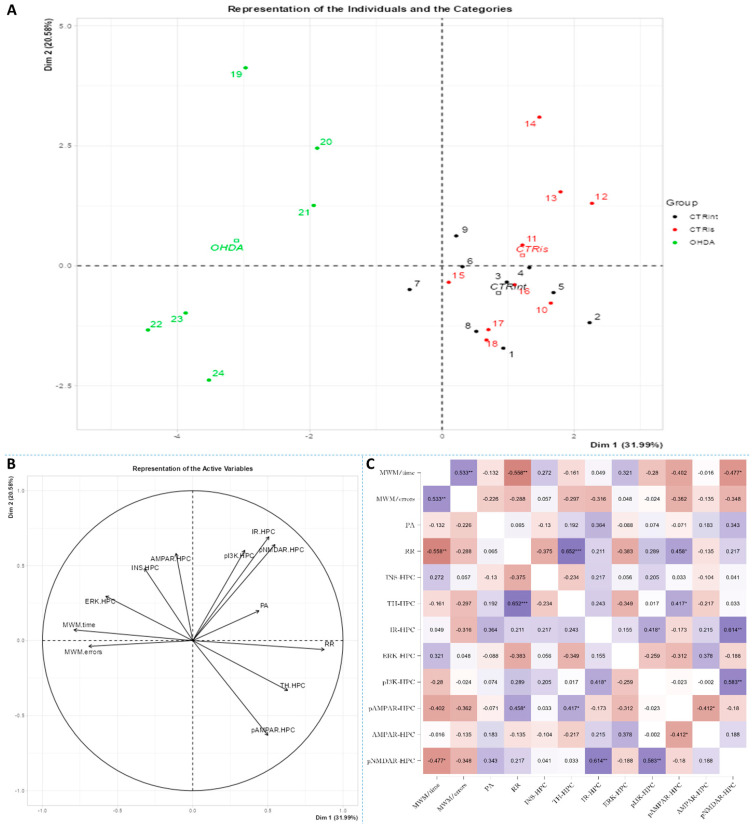
Principal component analysis and Spearman’s rank correlation of observed parameters in the hippocampus 3 months after intrastriatal administration of 6-OHDA. Hippocampal (HPC) principal component analysis is represented as a biplot (**A**) with coordinates of individual animals in respect to the 1st and 2nd dimensions (principal components) and a biplot graph of the variables’ contributions to the 1st and the 2nd principal component (**B**). Groups are represented in different colors: intact control (CTRint, black), sham-treated control (CTRis, red), animals treated intrastriatally with 6-hydroxydopamine (OHDA, green) (**A**). Spearman’s rank correlation of HPC parameters is represented as Spearman’s rho heatmap (**C**) with statistically significant correlations indicated with * *p* < 0.05, ** *p* < 0.001, *** *p* < 0.001. Dim 1-1st dimension; Dim 2-2nd dimension; MWM-Morris Water Maze; PA-Passive Avoidance; RR-Rotarod; INS-insulin; TH-tyrosine hydroxylase; IR-insulin receptor; ERK-extracellular signal-regulated kinase; pI3K-phosphoinositide 3-kinases; AMPAR-α-amino-3-hydroxy-5-methyl-4-isoxazolepropionic acid receptor; NMDAR-N-methyl-D-aspartate receptor.

**Figure 6:**
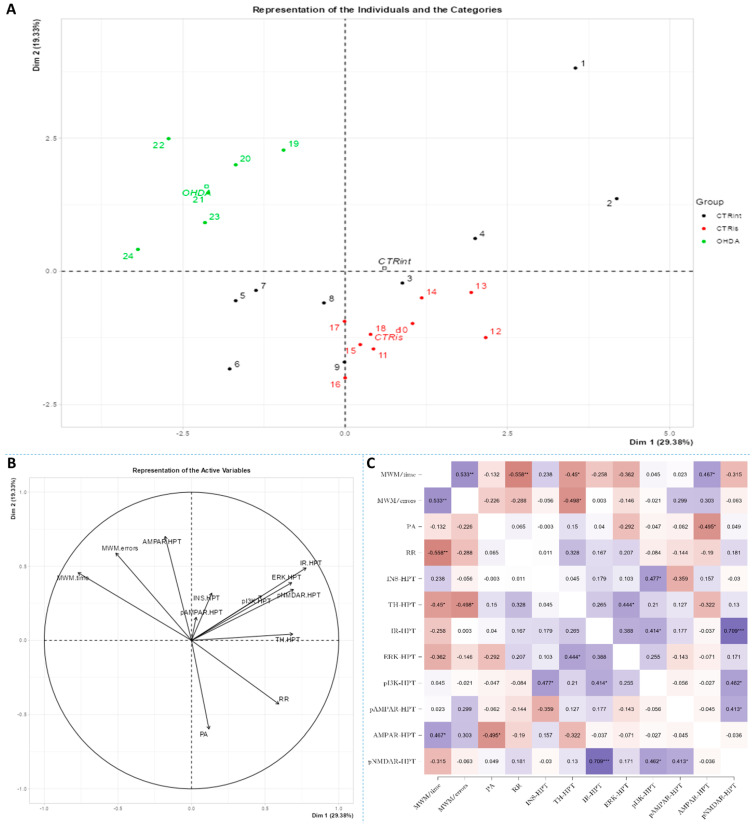
Principal component analysis and Spearman’s rank correlation of observed parameters in the hypothalamus 3 months after intrastriatal administration of 6-OHDA. Hypothalamic (HPT) principal component analysis is represented as a biplot (**A**) with coordinates of individual animals in respect to the 1st and 2nd dimensions (principal components) and a biplot graph of the variables’ contributions to the 1st and the 2nd principal component (**B**). Groups are represented in different colors: intact control (CTRint, black), sham-treated control (CTRis, red), animals treated intrastriatally with 6-hydroxydopamine (OHDA, green) (**A**). Spearman’s rank correlation of HPT parameters is represented as Spearman’s rho heatmap (**C**) with statistically significant correlations indicated with * *p* < 0.05, ** *p* < 0.001, *** *p* < 0.001. Dim 1-1st dimension; Dim 2-2nd dimension; MWM-Morris Water Maze; PA-Passive Avoidance; RR-Rotarod; INS-insulin; TH-tyrosine hydroxylase; IR-insulin receptor; ERK-extracellular signal-regulated kinase; pI3K-phosphoinositide 3-kinases; AMPAR-α-amino-3-hydroxy-5-methyl-4-isoxazolepropionic acid receptor; NMDAR-N-methyl-D-aspartate receptor.

**Figure 7:**
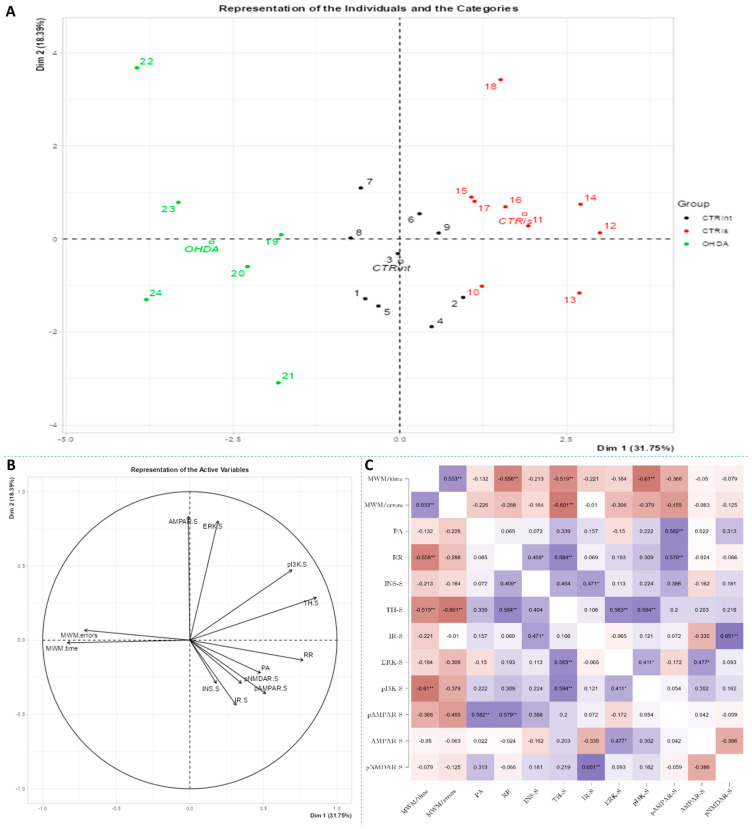
Principal component analysis and Spearman’s rank correlation of observed parameters in the striatum 3 months after intrastriatal administration of 6-OHDA. Striatal (S) principal component analysis is represented as a biplot (**A**) with coordinates of individual animals in respect to the 1st and 2nd dimensions (principal components) and a biplot graph of the variables’ contributions to the 1st and the 2nd principal component (**B**). Groups are represented in different colors: intact control (CTRint, black), sham-treated control (CTRis, red), animals treated intrastriatally with 6-hydroxydopamine (OHDA, green) (**A**). Spearman’s rank correlation of S parameters is represented as Spearman’s rho heatmap (**C**) with statistically significant correlations indicated with * *p* < 0.05, ** *p* < 0.001. Dim 1-1st dimension; Dim 2—2nd dimension; MWM-Morris Water Maze; PA-Passive Avoidance; RR-Rotarod; INS-insulin; TH-tyrosine hydroxylase; IR-insulin receptor; ERK-extracellular signal-regulated kinase; pI3K-phosphoinositide 3-kinases; AMPAR-α-amino-3-hydroxy-5-methyl-4-isoxazolepropionic acid receptor; NMDAR-N-methyl-D-aspartate receptor.

Spearman’s rank correlation was performed to analyze monotonic associations of the behavioral results’ parameters and measured parameters in the HPC (Figure 5C), HPT (Figure 6C) and S (Figure 7C). Regarding the behavioral parameters, there was a strong positive correlation between time needed to find platform (MWM/time) and non-target entries (MWM/errors) (ρ = +0.533, *p* = 0.007) and strong negative correlation between MWM/time and Rotarod (RR) results (ρ = −0.558, *p* = 0.008) (Figure 5, Figure 6 and Figure 7; Supplement S2). In all of the observed regions, there was a strong positive correlation between pNMDAR and IR levels (ρ = +0.614 in HPC, *p* = 0.005/Figure 5C/; ρ = +0.709 in HPT, *p* < 0.001/Figure 6C/; ρ = +0.651 in S, *p* = 0.001/Figure 7C/). In both HPC (Figure 5C) and S (Figure 7C), there was a strong positive correlation between TH levels and RR results (ρ = +0.652, *p* < 0.001 and +0.584. *p* = 0.003, respectively). In the HPC, pNMDAR was found positively correlated with pI3K (ρ = +0.583, *p* = 0.008). In the S (Figure 7C), TH was found strongly negatively correlated with MWM/errors (ρ = −0.601, *p* = 0.002), while positively with ERK (ρ = +0.563, *p* = 0.005) and pI3K (ρ = +0.594, *p* = 0.003). Additionally, in the S (Figure 7C), pAMPAR was found positively correlated with PA (ρ = +0.582, *p* = 0.009) and RR (ρ = +0.579, *p* = 0.009), and pI3K was found negatively correlated with MWM/time (ρ = −0.610, *p* = 0.002). Other moderate associations (ρ =0.4–0.53) are indicated in Figure 5C, Figure 6C and Figure 7C and Supplement S2. Principal component analysis was used for multivariate exploration separately in the HPC (Figure 5), HPT (Figure 6) and S (Figure 7). Plots in Figure 5A, Figure 6A and Figure 7A represent coordinates of individuals in respect to the first and second PC for each region (HPC, HPT and S) and behavioral data. In the HPC, the first two components explain 31.99% and 20.58% of the variance, respectively, with RR, MWM/time and errors, TH and ERK being the largest contributors to the first PC and IR, pAMPAR and pNMDAR to the second one (Figure 5A,B; Supplement S2). Clustering of the groups, OHDA vs. CTRint and CTRis was most prominent in respect to the first PC in the HPC (Figure 5A). In the HPT, two components explained 29.38% and 19.33% of the variance, with MWM/time, IR, pNMDAR, TH and ERK being the largest contributors to the first PC and AMPAR and PA to the second one (Figure 6A,B; Supplement S2). The 6-OHDA treatment showed the most pronounced effect in respect to the both first and second PC in the HPT (Figure 6A). In the S, the first two components explain 31.75% and 18.39% of the variance with MWM/time and errors, TH, pI3K and RR as the largest contributors to the first PC and IR and AMPAR and ERK to the second one (Figure 7A,B; Supplement S2). Both intrastriatal administrations 6-OHDA (OHDA) and vehicle (CTRis) had pronounced opposite effects in respect to the first PC (Figure 7A).

## 4. Discussion

Bilateral intrastriatal 6-OHDA administration triggers dopaminergic degeneration in both hemispheres, which is important for the appearance of the entire spectrum of behavioral symptoms of the disease [30,37,38,39]. Given that dopamine is a key neurotransmitter involved in motivation, stimulus-reward learning process and preserving neuronal plasticity, its malfunction can lead to changes in synaptic transmission as well as cognitive disorders [40]. The occurrence of memory and learning disorders is expected and registered in animals treated with 6-OHDA toxin [41,42]. In addition, the 6-OHDA model displays the broader spectrum of non-motor symptoms (emotional and cognitive deficit) in comparison to the genetic PD model [41].

The results confirm that the bilateral 6-OHDA injection causes motor symptoms characteristic for PD [31], as well as dysfunctions in spatial–visual memory and learning (Figure 1). Many authors associate the described changes with a decrease in the number of TH-positive neurons in the SNpc and striatum [43]. As TH is crucial in the synthesis of dopamine in the brain [44,45], it is expected that the depletion of dopaminergic neurons will lead to a decrease in TH positive neurons in the observed parts of the brain. Moreover, dopaminergic signaling has important role in the hippocampus-dependent learning and memory [46] and the hypothalamic control of energy metabolism [47] and they can receive dopamine projections from the ventral tegmental area (VTA) and SNpc [48,49,50,51]. Reduction in the TH positive signal was seen in all of the observed regions (HPC, HPT, S and CTX; Figure 2), and dopaminergic nuclei in the substantia nigra were found preserved 3 months after 6-OHDA bilateral injection, pointing towards incomplete depletion of dopaminergic cells. Tadaiesky and colleagues noticed, in SN, a cell number decrement with 6-OHDA bilateral injection (12 µg per injection) even 1 week after surgery, although the reduction persisted until the third week, it was diminished [25], suggesting a partial depletion and time-dependent recuperation of dopaminergic neurons in SN. There is a possibility that due to the longer time after 6-OHDA treatment and/or lower 6-OHDA dosage than in previous research [25,26], preservation of dopaminergic nuclei observed 3 months after 6-OHDA treatment might occur. Moreover, as we have already mentioned in our preprint, there was a pronounced drop-out rate in the 6-OHDA-treated group of animals (57% fatality rate) which may have introduced attrition bias—e.g., the animals with the most pronounced response to the 6-OHDA administration, and possibly more rapid spreading of the 6-OHDA-induced damage from the striatal terminals to SN [31]. The levels of TH in hypothalamus, in comparison to other observed regions, were found the most preserved, showing only a slight decrement. There was an increase in TH activity in S of sham operated group, suggesting a compensational response due to invasive procedure of intrastriatal application. PCA and correlation analysis were used to determine in which brain region the TH signal could be associated with behavioral changes (Figure 5, Figure 6 and Figure 7). Strong correlation between motoric performance and TH signal in HPC and S suggests involvement of dopaminergic signaling on motoric performance not only in striatum, as expected, but also in HPC. In addition, TH, RR and MWM are one of the most important parameters that can explain the difference between control groups and OHDA in HPC and S. It seems that spatial learning and memory does not correlate with dopaminergic signaling, but only in HPC, while there was some correlation in both HPT and S suggesting better performance in MWM with increment of TH levels. In spatial navigation tasks, the role of the hippocampus has been classically juxtaposed with the role of the dorsal striatum, the latter of which has been characterized as a system important for implementing stimulus–response and action–outcome associations [52]. It is possible that dopamine depletion in the striatum induced by 6-OHDA administration is responsible for learning and memory deficit, and that dopamine does not play an important role of aforementioned memory in HPC. There is also a possibility of simple direct action of 6-OHDA toxicity in HPC, which caused reduction in TH levels or reduced input of dopaminergic projections to this brain region. In addition, dopamine levels were found not altered in HPC after 6-OHDA treatment in the research of Tadaiesky and colleagues [25], and they also suggest that HPC is not involved in the behavioral impairments. However, decrement of TH can also be found in HPC after 6-OHDA injection in SN [53], this type of lesioning (in SN) is sometimes not adequate to develop non-motor symptoms. Striatal 6-OHDA injections are better at reproducing the early stages of PD, so this approach is well suited to the study of non-motor symptoms, since these ailments often appear during the prodromal phase of PD [54], and once again indicate the importance of striatum in spatial learning and memory. The administration of 6-OHDA leads to damaging noradrenergic neurons as well, which can be associated with both central (cognitive impairment, depression, anxiety, olfactory deficit) as well as peripheral (cardiovascular, gastrointestinal) non-motor symptoms [55]. Interestingly, only in HPT, the parameter measuring fear-conditioned memory, in addition to MWM, is one of the important ones that explains the difference between CTR and OHDA groups. Research on fear conditioning has traditionally focused on brain areas such as the amygdala and hippocampus, in contrast, the role of the hypothalamus has remained relatively underexplored. Recently, Concetti and colleagues found that inappropriate, learning-resistant fear results from disruption of brain components: hypothalamic melanin-concentrating hormone-expressing neurons [56], confirming the importance of HPT in memory disorders. In addition, sleep disorders appear in the early stages of PD and this can be due to degeneration of orexinergic neurons in HPT [57], as it was also noted in rat PD model after striatal 6-OHDA injection [58].

The importance of insulin in the metabolism of glucose in the brain has gained attention in the last few decades through research into the pathogenesis of neurodegenerative diseases such as AD and PD. Insulin via the PI3K/Akt signaling pathway enhances long-term memory in hippocampus-related tasks, reduces alpha-synuclein accumulation and neurotoxicity and reduces neuroinflammation and apoptosis [59]. In addition, insulin normalizes the production and functionality of dopamine and ameliorates motor impairments in 6-OHDA-induced rat PD models [59]. Although insulin resistance is increasingly associated with PD, this mechanism has not yet been fully investigated. Interestingly, PD patients show increased autoimmune reactivity to insulin that may reflect the neurodegenerative brain-damaging processes and impaired insulin homeostasis occurring in PD [60]. Moreover, in PD patients, the death of dopaminergic neurons in the SNpc is often associated with a marked loss of IR mRNA and an increased level of IRS phosphorylation at serine residues, with an inhibitory effect on insulin signaling and subsequently increased insulin resistance [61]. Signs of insulin resistance were found in the rat striatum 6 weeks following the unilateral administration of 6-OHDA into the MFB [12]. In rats with severe depletion of dopamine (90–99%), the most prominent signs in the brain were increased serine phosphorylation of IR substrate-2 (IRS2) and decreased phosphorylation of AKT as well as reduced expression of GSK-3α, while no significant changes in peripheral glucose and insulin were found. While unilateral intrastriatal 6-OHDA administration has been shown to affect serum insulin levels (early increase in glucose tolerance test) and striatal insulin signaling, it has not induced peripheral insulin resistance, at least not at the 6-week post-lesion time point. [62]. However, induction of peripheral insulin resistance by a diet high in fat may lower the threshold for developing experimental PD following 6-OHDA treatment [63]. Insulin binding to IR was found decreased by 25% in the arcuate nucleus 7 days after 6-OHDA icv treatment [64], but the dose that was used was fairly high. A dysfunctional IR signaling cascade in the 6-OHDA model was reported in the striatum; reduction in PI3K and AKT phosphorylation was found after 2 [65] and 6 weeks [12]. Similarly, increased levels of phosphorylated IRS at serine residues, the marker of insulin resistance, are seen in the dopamine-depleted striatum, in the 6-OHDA toxin model of PD and transgenic mice overexpressing alpha-synuclein [12,66]. Contrary to these findings, our results do not show marked changes in levels of proteins in insulin cascade pathway 3 months after 6-OHDA administration, with a slight tendency for insulin concentration decrement in the hypothalamus and striatum (Figure 3). As we have already mentioned, this discrepancy could be related to lower dose administration and partial depletion of dopaminergic neurons. There is a possibility that changes in the insulin signaling pathway protein expression would be observed at earlier and/or later time points after 6-OHDA administration than the time point used in this study, where a compensational response could happen. Conversely, PCA and correlation analysis suggested involvement of IR levels in hypothalamic CTR and OHDA distinguishment: IR being the largest contributor to the first dimension (Figure 6, Supplement S2), suggesting a connection between dopaminergic depletion (induced primarily in striatum) with IR signaling in HPT. As was expected, the strong positive correlation in all of the observed regions was found between levels of IR and pNMDAR, confirming their signaling pathway interconnection [67,68]. Interestingly, in the striatum, TH was found positively correlated with ERK and pI3K, suggesting the importance of MAPK/ERK and PI3K/AKT signaling pathways in catecholamine synapses not necessarily connected with IR pathway (Figure 7). Summarizing the results, we can assume that disturbed dopamine signaling may be, to a lesser extent, transduced to insulin signaling and its downstream targets, as a secondary, collateral damage [55] such as the one seen in HPT, but it probably depends on the 6-OHDA dose and the location of administration. In addition, intranasal insulin improves mitochondrial function, alleviates motor deficits and protects against substantia nigra dopaminergic neuronal loss in 6-OHDA rat model [69,70], suggesting a neuroprotective insulin action.

Glutamate, as an important central neurotransmitter, is closely related to the occurrence and development of PD [16], and impaired glutamate homeostasis in the striatum is emerging as a key feature of PD pathology [19]. It has been proven that glutamate AMPA receptors participate in the modulation of neuronal excitability and long-term synaptic plasticity [71] and are associated with altered neurotransmission in PD [72]. By analyzing the protein levels of the AMPA receptor and its phosphorylated form, we observed significant changes in all three regions of the brain. A decrease in phosphorylated AMPA receptor in the HPC and S was observed 3 months after bilateral intrastriatal administration of 6-OHDA, which indicates reduced glutamate signaling in these brain regions. PCA analysis indicated the importance of glutamate signaling in HPT, where increased pNMDAR levels and decreased AMPAR levels distinguish control animals from OHDA group. If we include the finding that reduced pNMDAR levels are correlated with reduced IR levels in the HPT of OHDA group, increased AMPAR could be a compensational response to decreased glutamatergic signaling. Interestingly, increased levels of pAMPAR in S are associated with better results in RR and PA tests (Figure 7). Previous studies have shown that α-synuclein is closely associated with glutamate excitotoxicity and that the aforementioned α-synuclein increases the release of glutamate [16]. Additionally, overexpression of α-synuclein increases the phosphorylation of NMDARs, and some studies indicate that α-synuclein can also enhance glutamate excitotoxicity by accelerating AMPAR signaling [16]. The 6-OHDA model shares a common failing with many other animal models of PD as it does not lead to the formation of the pathological hallmark of PD, the Lewy body, that contain ubiquitinated proteins such as α-synuclein [73]; therefore, the change in glutamate receptor levels in our research, especially AMPA receptors, is associated with dopaminergic degeneration seen as positive correlation between TH and pAMPAR levels. In their work, He and his co-workers [74] showed that direct administration of 6-OHDA in the SNpc reduces the expression of AMPA receptors, including its subunits GluR1, GluR2 and GluR3, but not GluR4 and this could be related to the reduction in TH positive neurons, considering that in the study the expression of the AMPA receptor, GluR1 subunit, was decreased together with the appearance of lesions and the reduction in TH positive neurons after the administration of 6-OHDA [74]. Recent studies suggest that selective potentiators of AMPA receptors may be useful for protection against SNc degeneration and motor deficits after establishment of SNc lesion caused by 6-OHDA in rats [75]. There is a possibility that the decrement of pAMPAR levels is a consequence of synapse weakening following glutamate excitotoxicity that occurred earlier after dopaminergic degeneration by 6-OHDA injection. Dopaminergic and glutamatergic neurons are interconnected. The striatum circuitry is mainly composed of inhibitory spiny projection neurons (SPNs) that receive DA innervation from SNpc and glutamatergic inputs from the cerebral cortex and thalamus. The coordinated activity of these pathways becomes impaired in PD due to the progressive loss of nigral DA neurons. A decreased stimulation of the DA receptors affects the direct pathway (striatopallidal neurons project to neurons of the internal segment of the globus pallidus directly) leading to a reduced inhibition of the output signals while the lack of DA receptor-mediated stimulation of the indirect pathway (striatopallidal neurons communicate with the external segment of the globus pallidus and the subthalamic nucleus (STN) through glutamate release) results in a disinhibition of the STN causing a glutamatergic overstimulation of the output signals [19]. Excessive activation of NMDARs induces excessive influx of Ca2+ and further exacerbates ROS levels and oxidative damage. Overactivation of AMPARs induces Na+ overload, which leads to increased cellular permeability and results in cellular swelling and neuronal death [16]. If this was the course of events, we should also see a decrease in the level of total receptors AMPARs, as well as NMDARs, but that is not the case. Reduced pAMPAR levels can be a sign of synapse weakening. In HPC, long term depression is dependent on NMDARs and its induction can lead to the depression of synaptic strength via removal of AMPARs [71]. This can lead to exaggeration of motoric and cognitive deficit, especially since there is a correlation between striatal pAMPAR levels and cognitive and motor performance in this research. In addition, sham operation caused decrement of insulin concentration and pAMPAR level in HPC compared to intact control, which indicates chronic changes (3 months after the surgery) after striatal syringe lesioning.

To conclude, bilateral intrastriatal administration of 6-OHDA can induce cognitive deficit, beside motoric one and substantial reduction in TH levels in the brain, but with incomplete depletion of dopaminergic SN cells. This partial depletion could be due to the too low 6-OHDA dose or time-dependent recuperation of dopaminergic neurons in SN. The effects of 6-OHDA injection on rodent brain are known in general and the novelty of this research is that it provides the novel findings on the region-specificity in the particular signaling pathways (comparison between insulin and glutamate signaling) and the correlation of these changes with behavioral patterns and dopaminergic depletion, which originated in the striatum. Even though the levels of proteins involved in insulin signaling were not found markedly changed, PCA and correlation analysis suggested some association such as in the case of: (a) IR being one of the largest contributors to the first PC in HPT (CTRs and OHDA group distinguishment), suggesting a connection between dopaminergic depletion with IR signaling in HPT; and (b) a positive correlation between striatal TH and ERK and pI3K. Further studies are needed to evaluate the involvement of insulin signaling, especially regarding transcriptional changes and their correlation to protein expression. The most pronounced change in glutamatergic receptor levels was the decrement of pAMPAR in S and HPC and its positive association with better performance in motoric and cognitive tests. Decrement of glutamatergic signaling can be due to synapse weakening and can lead to exaggeration of motoric and cognitive deficit. Further studies are needed to clarify the exact role and the involvement of striatal glutamatergic signaling in motoric and cognitive functions.

## Data Availability

The datasets generated during and/or analyzed during the current study are available in the Mendeley Data repository (accessed on 9 January 2023), https://data.mendeley.com/drafts/9dv35g4sjg (Knezovic, Ana; Piknjac, Marija; Osmanovic Barilar, Jelena; Babić Perhoč, Ana; Virag, Davor; Homolak, Jan; Salkovic-Petrisic, Melita (2023), “Association of cognitive deficit with glutamate and insulin signaling in a rat model of Parkinson’s disease”, Mendeley Data, V1, doi: 10.17632/9dv35g4sjg.1).

## Supplementary Materials

The following supporting information can be downloaded at: https://www.mdpi.com/article/10.3390/biomedicines11030683/s1, Supplement S1—Time spent in search for the platform and non-target entries during the probe MWM trial; Supplement S2—PCA analysis and correlation matrix.
