# Supplementary material for: Association of Cognitive Deficit with Glutamate and Insulin Signaling in a Rat Model of Parkinson’s Disease
Source: Biomedicines. 2023 Feb 23;11(3):683. doi: 10.3390/biomedicines11030683 (PMC10045263; doi:10.3390/biomedicines11030683)
Supplement: Supplementary file 1 [file biomedicines-11-00683-s001.zip › biomedicines-2175969-supplementary.pdf]

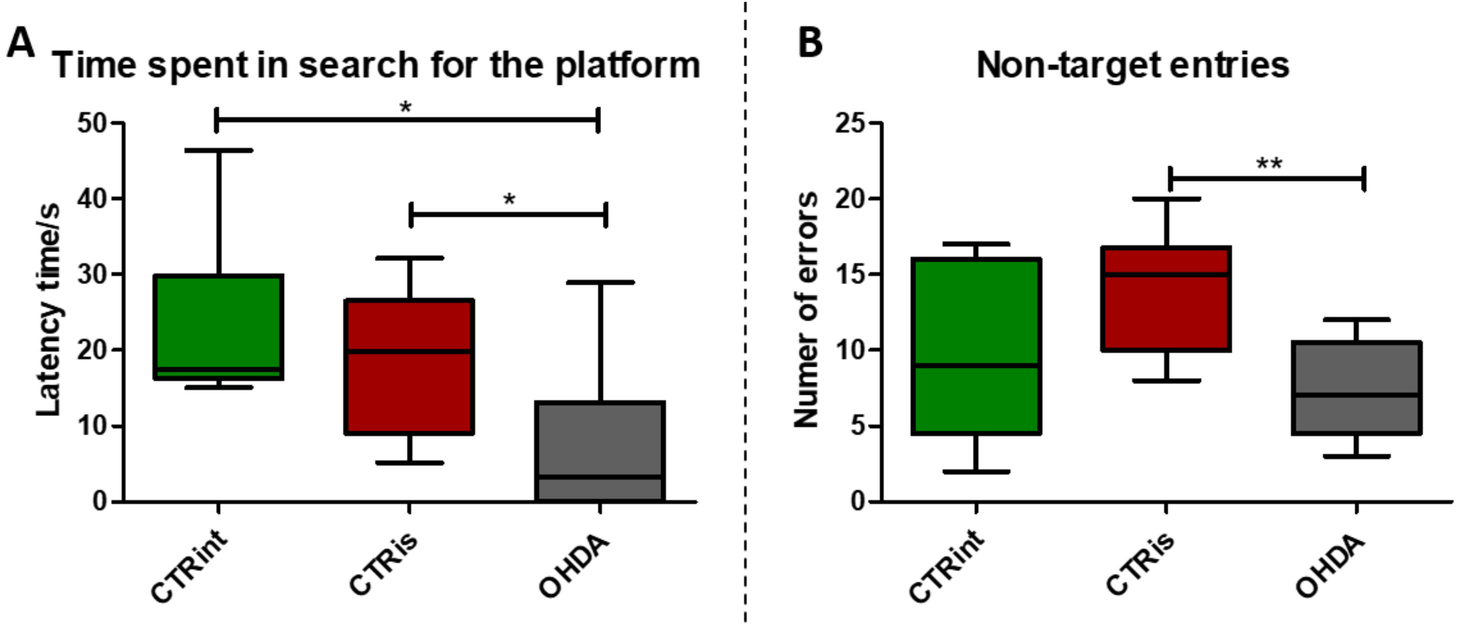

A
Time spent in search for the platform
Latency time/s
0
10
20
30
40
50
*
*
CTRint
CTRis
OHDA
B
Non-target entries
Numer of errors
0
5
10
15
20
25
**
CTRint
CTRis
OHDA

# Results of the Principal Component Analysis

## Representation of the Individuals (and the Categories)

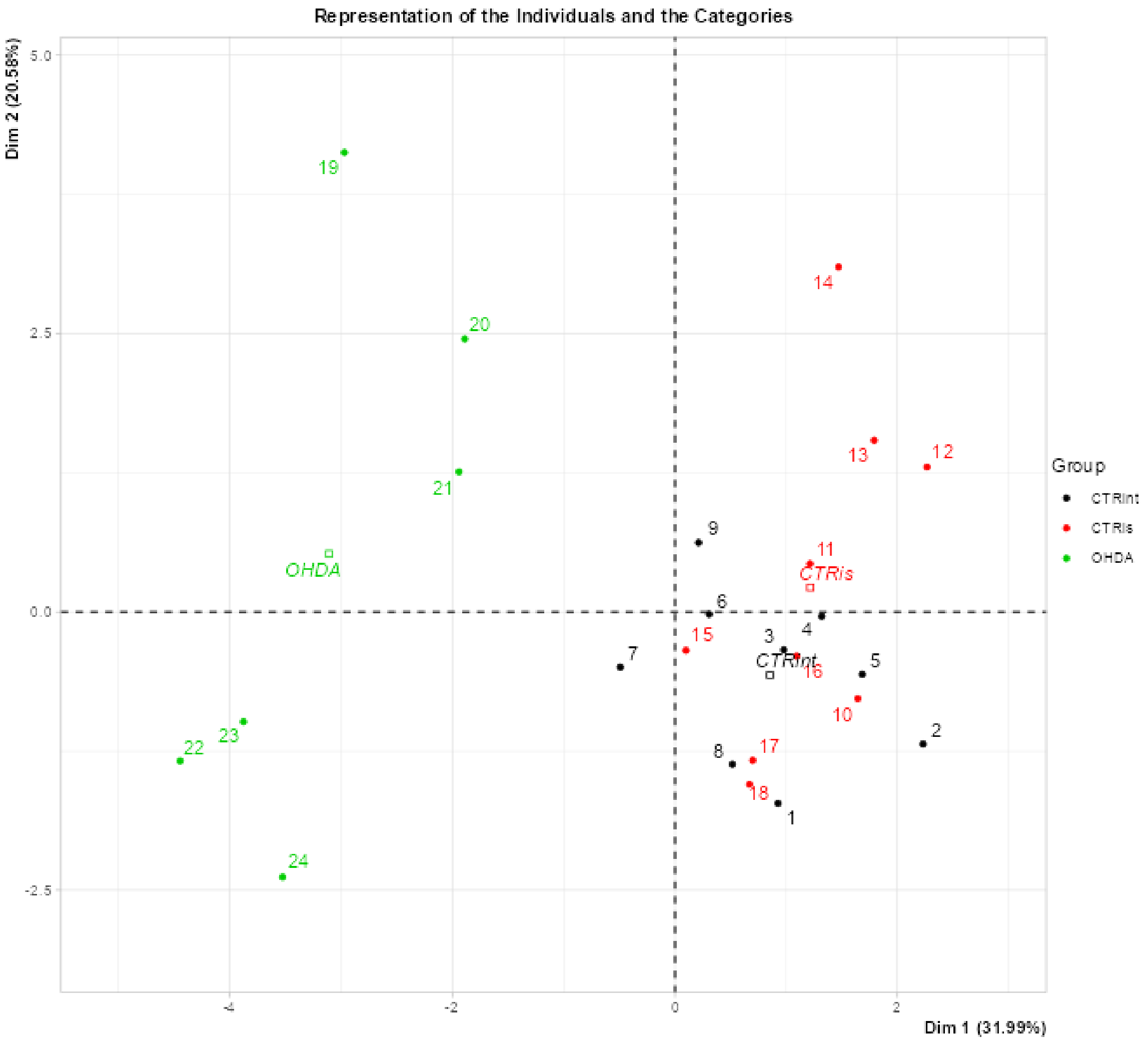


## Representation of the Variables

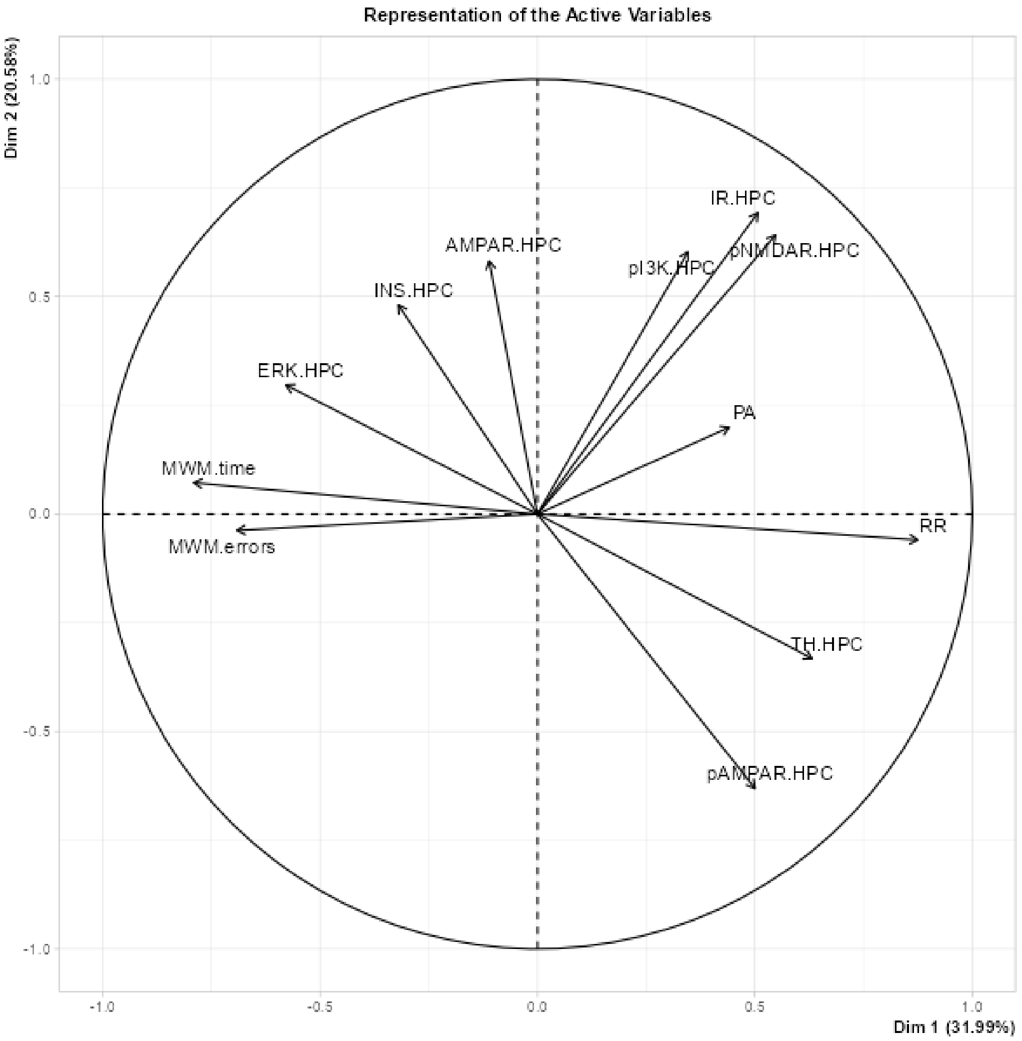


## Eigenvalue Decomposition

Eigenvalue and (Cumulative) Percentage of Variance

| | Eigenvalue | % of the variance | Cumulative % |
|---|---|---|---|
| Dim. 1 | 3.8389 | 31.991 | 32.0 |
| Dim. 2 | 2.4696 | 20.580 | 52.6 |
| Dim. 3 | 1.4273 | 11.895 | 64.5 |
| Dim. 4 | 1.0166 | 8.472 | 72.9 |
| Dim. 5 | 0.9556 | 7.963 | 80.9 |
| Dim. 6 | 0.7008 | 5.840 | 86.7 |
| Dim. 7 | 0.4801 | 4.001 | 90.7 |
| Dim. 8 | 0.3677 | 3.064 | 93.8 |
| Dim. 9 | 0.3103 | 2.586 | 96.4 |
| Dim. 10 | 0.2307 | 1.922 | 98.3 |
| Dim. 11 | 0.1562 | 1.302 | 99.6 |
| Dim. 12 | 0.0462 | 0.385 | 100.0 |

## Automatic Description of the Axes

```
$Dim.1
$quanti
           correlation   p.value
RR              0.8750 2.226e-08
TH.HPC          0.6319 9.261e-04
pNMDAR.HPC      0.5474 5.632e-03
IR.HPC          0.5072 1.141e-02
pAMPAR.HPC      0.5006 1.271e-02
PA              0.4411 3.096e-02
ERK.HPC        -0.5786 3.059e-03
MWM.errors     -0.6912 1.841e-04
MWM.time       -0.7905 4.303e-06
```

```
$quali
          R2   p.value
Group 0.8441 3.359e-09

$category
            Estimate   p.value
Group=CTRis    1.562 1.734e-02
Group=OHDA    -2.761 3.771e-10

attr(,"class")
[1] "condes" "list"

$Dim.2
$quanti
           correlation   p.value
IR.HPC          0.6925 0.0001769
pNMDAR.HPC      0.6407 0.0007427
pI3K.HPC        0.6028 0.0018241
AMPAR.HPC       0.5818 0.0028608
INS.HPC         0.4807 0.0174318
pAMPAR.HPC     -0.6310 0.0009450

attr(,"class")
[1] "condes" "list"

$Dim.3
$quanti
          correlation   p.value
AMPAR.HPC      0.5479 0.0055820
PA             0.4678 0.0211452
pI3K.HPC      -0.5231 0.0087220
INS.HPC       -0.6376 0.0008035

attr(,"class")
[1] "condes" "list"

$Dim.4
$quanti
           correlation p.value
PA              0.4425 0.03036
MWM.errors      0.4366 0.03290
MWM.time        0.4253 0.03830

attr(,"class")
[1] "condes" "list"

$Dim.5
$quanti
        correlation p.value
ERK.HPC      0.4384 0.03214

$quali
          R2  p.value
Group 0.4163 0.003507

$category
             Estimate  p.value
Group=CTRint   0.8132 0.001109
Group=CTRis   -0.6118 0.011893

attr(,"class")
[1] "condes" "list"
```

## Variable Tables

Contributions Table

| | Dim.1 | Dim.2 | Dim.3 | Dim.4 | Dim.5 |
|---|---|---|---|---|---|
| INS.HPC | 2.665 | 9.3547 | 28.4829 | 1.5906 | 11.3070 |
| TH.HPC | 10.400 | 4.4784 | 2.9792 | 13.6100 | 10.6091 |
| IR.HPC | 6.701 | 19.4180 | 0.1332 | 1.2409 | 16.0273 |
| ERK.HPC | 8.720 | 3.5625 | 7.3990 | 13.8677 | 20.1085 |
| pI3K.HPC | 3.125 | 14.7123 | 19.1690 | 0.1258 | 4.4101 |
| pAMPAR.HPC | 6.529 | 16.1243 | 4.2322 | 3.5130 | 5.3963 |
| AMPAR.HPC | 0.321 | 13.7065 | 21.0286 | 8.9999 | 4.2137 |
| pNMDAR.HPC | 7.805 | 16.6244 | 0.0194 | 1.2240 | 2.5776 |
| MWM.time | 16.277 | 0.2079 | 0.7950 | 17.7891 | 10.2073 |
| MWM.errors | 12.444 | 0.0585 | 0.2821 | 18.7536 | 14.9686 |
| PA | 5.068 | 1.6082 | 15.3341 | 19.2621 | 0.1240 |
| RR | 19.944 | 0.1444 | 0.1451 | 0.0233 | 0.0506 |

# Principal Component Analysis

## Representation of the Individuals (and the Categories)

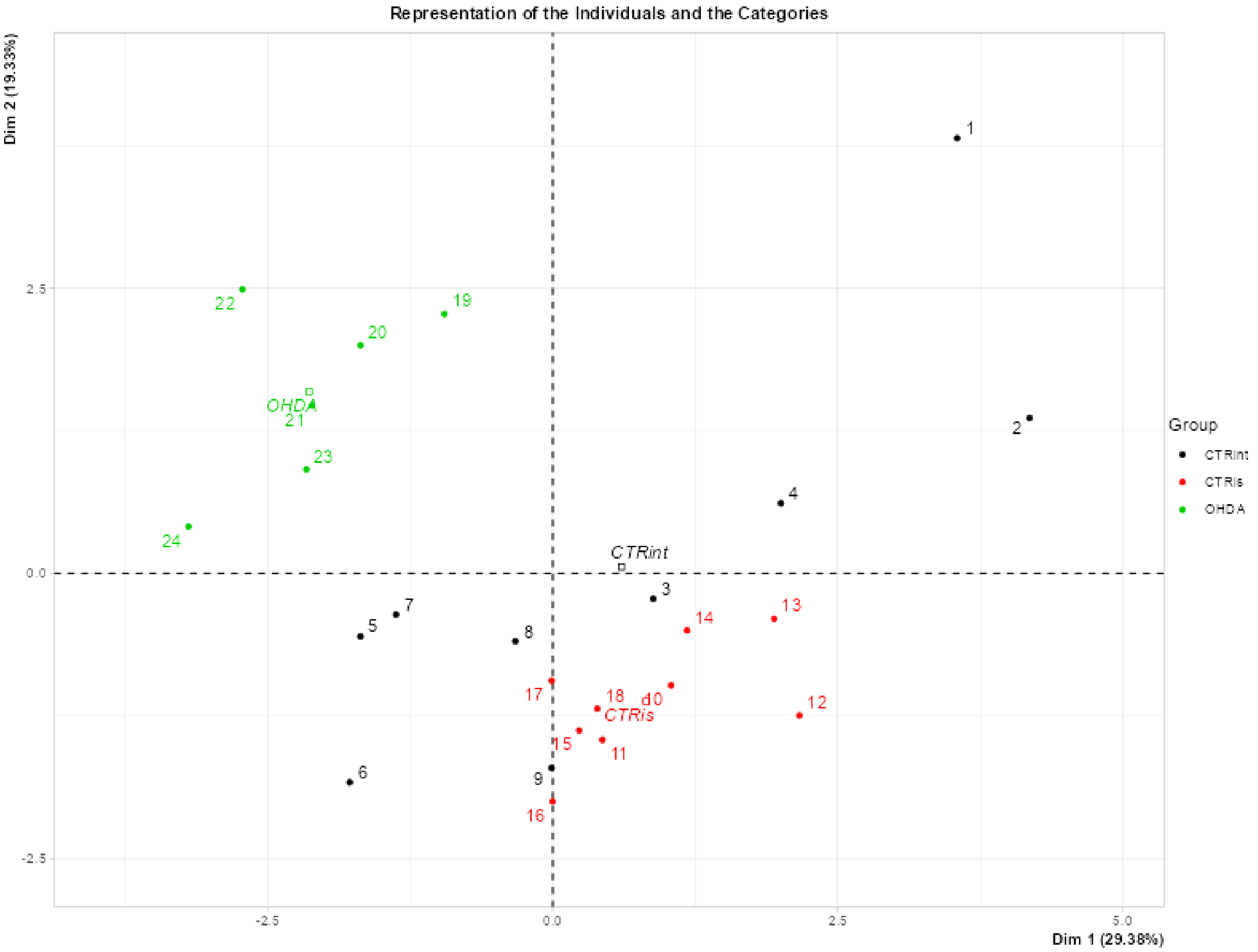


## Representation of the Variables

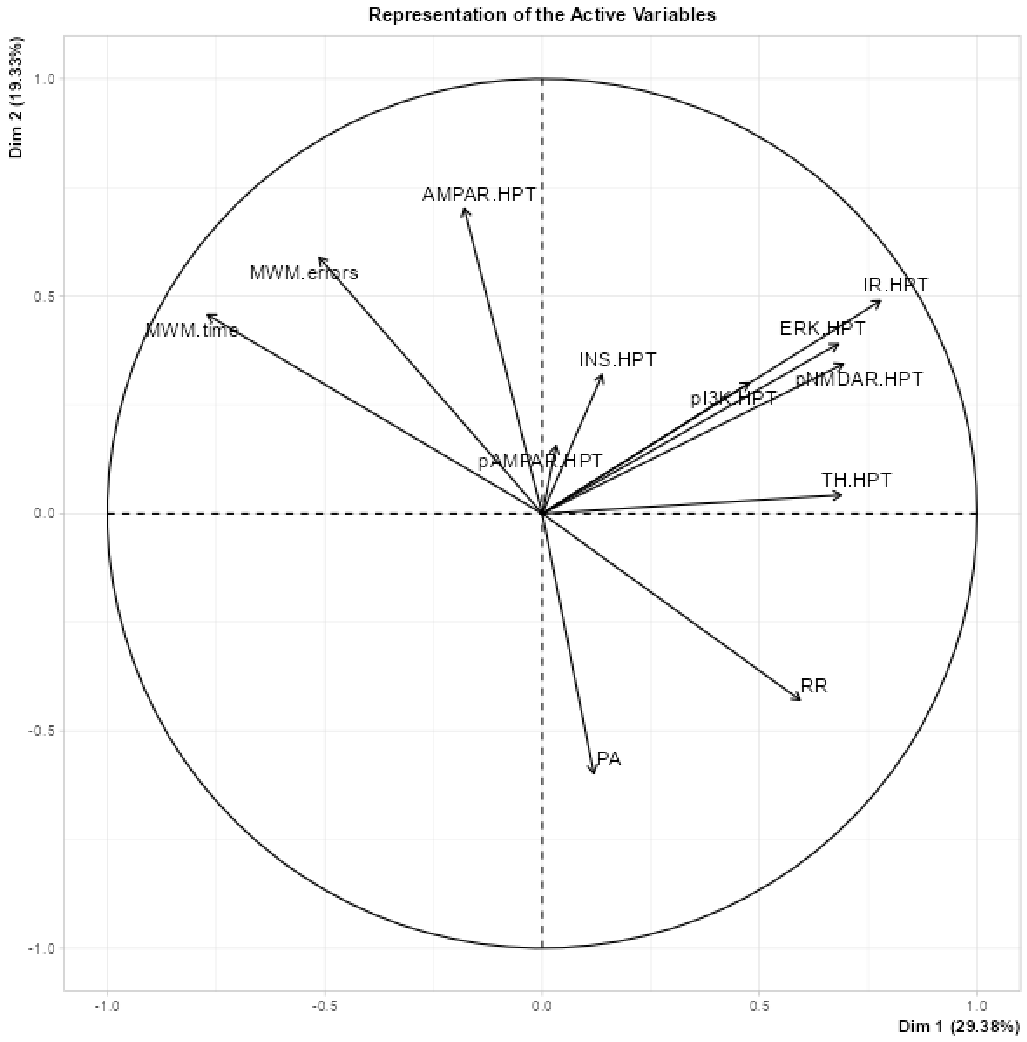


## Eigenvalue Decomposition

Eigenvalue and (Cumulative) Percentage of Variance

| | Eigenvalue | % of the variance | Cumulative % |
|---|---|---|---|
| Dim. 1 | 3.5255 | 29.379 | 29.4 |
| Dim. 2 | 2.3196 | 19.330 | 48.7 |
| Dim. 3 | 1.7189 | 14.324 | 63.0 |
| Dim. 4 | 1.1776 | 9.813 | 72.8 |
| Dim. 5 | 0.8999 | 7.499 | 80.3 |
| Dim. 6 | 0.8048 | 6.706 | 87.1 |
| Dim. 7 | 0.4918 | 4.098 | 91.2 |
| Dim. 8 | 0.4397 | 3.664 | 94.8 |
| Dim. 9 | 0.3158 | 2.632 | 97.4 |
| Dim. 10 | 0.1432 | 1.193 | 98.6 |
| Dim. 11 | 0.1152 | 0.960 | 99.6 |
| Dim. 12 | 0.0480 | 0.400 | 100.0 |

## Automatic Description of the Axes

```
$Dim.1
$quanti
           correlation   p.value
IR.HPT          0.7783 7.523e-06
pNMDAR.HPT      0.6929 1.746e-04
TH.HPT          0.6882 2.016e-04
ERK.HPT         0.6808 2.503e-04
RR              0.5934 2.238e-03
pI3K.HPT        0.4763 1.864e-02
MWM.errors     -0.5135 1.027e-02
MWM.time       -0.7704 1.065e-05
```

```
$quali
          R2  p.value
Group 0.4336 0.002556

$category
           Estimate  p.value
Group=OHDA   -1.898 0.000492

attr(,"class")
[1] "condes" "list"

$Dim.2
$quanti
           correlation   p.value
AMPAR.HPT       0.7020 0.0001316
MWM.errors      0.5893 0.0024432
IR.HPT          0.4894 0.0152146
MWM.time        0.4572 0.0246759
RR             -0.4292 0.0363341
PA             -0.5980 0.0020270

$quali
          R2  p.value
Group 0.4771 0.001106

$category
            Estimate  p.value
Group=OHDA     1.416 0.001780
Group=CTRis   -1.298 0.003644

attr(,"class")
[1] "condes" "list"

$Dim.3
$quanti
           correlation   p.value
pAMPAR.HPT      0.8847 9.588e-09
pI3K.HPT       -0.4631 2.266e-02
INS.HPT        -0.7315 4.877e-05

attr(,"class")
[1] "condes" "list"

$Dim.4
$quanti
           correlation  p.value
PA              0.5776 0.003118
pI3K.HPT        0.5032 0.012185
pNMDAR.HPT      0.4133 0.044718
ERK.HPT        -0.4265 0.037672

attr(,"class")
[1] "condes" "list"

$Dim.5
$quanti
       correlation  p.value
TH.HPT      0.5785 0.003061

attr(,"class")
[1] "condes" "list"
```

## Variable Tables

Contributions Table

| | Dim.1 | Dim.2 | Dim.3 | Dim.4 | Dim.5 |
|---|---|---|---|---|---|
| INS.HPT | 0.5411 | 4.3951 | 31.12899 | 0.0760 | 12.7858 |
| TH.HPT | 13.4326 | 0.0783 | 3.09406 | 1.0753 | 37.1934 |
| IR.HPT | 17.1834 | 10.3258 | 0.45716 | 0.9721 | 1.2276 |
| ERK.HPT | 13.1485 | 6.5634 | 0.03459 | 15.4484 | 3.0400 |
| pI3K.HPT | 6.4336 | 3.8978 | 12.47867 | 21.5063 | 0.0313 |
| pAMPAR.HPT | 0.0285 | 1.0528 | 45.53466 | 3.0504 | 6.0485 |
| AMPAR.HPT | 0.9107 | 21.2454 | 0.09036 | 4.6275 | 15.2362 |
| pNMDAR.HPT | 13.6189 | 5.0967 | 1.95997 | 14.5042 | 14.2191 |
| MWM.time | 16.8341 | 9.0134 | 0.32499 | 1.8310 | 3.5986 |
| MWM.errors | 7.4802 | 14.9723 | 4.49977 | 4.1829 | 2.1746 |
| PA | 0.4000 | 15.4159 | 0.00509 | 28.3340 | 0.7155 |
| RR | 9.9883 | 7.9432 | 0.39169 | 4.3918 | 3.7294 |

# Results of the Principal Component Analysis

## Representation of the Individuals (and the Categories)

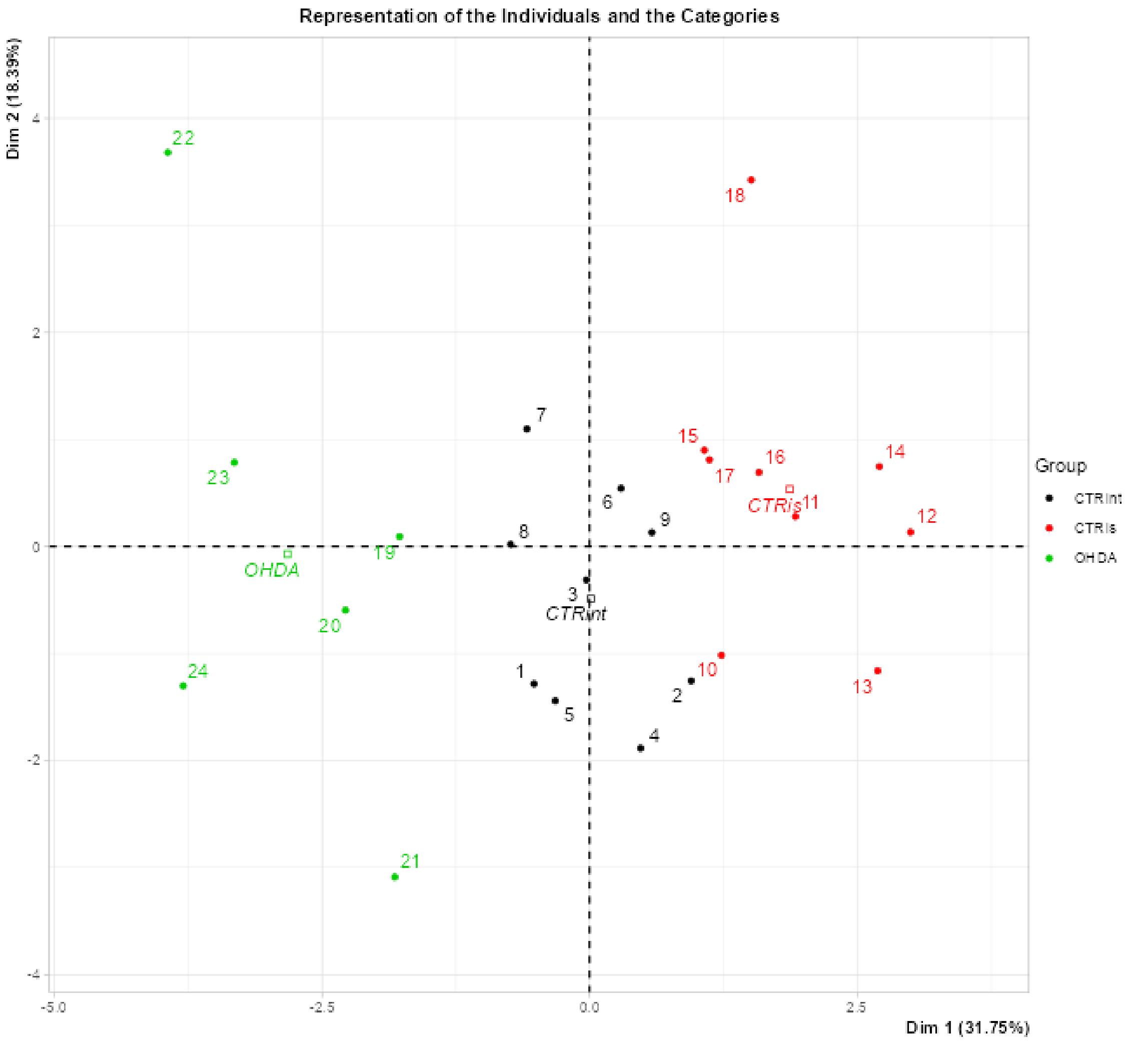


## Representation of the Variables

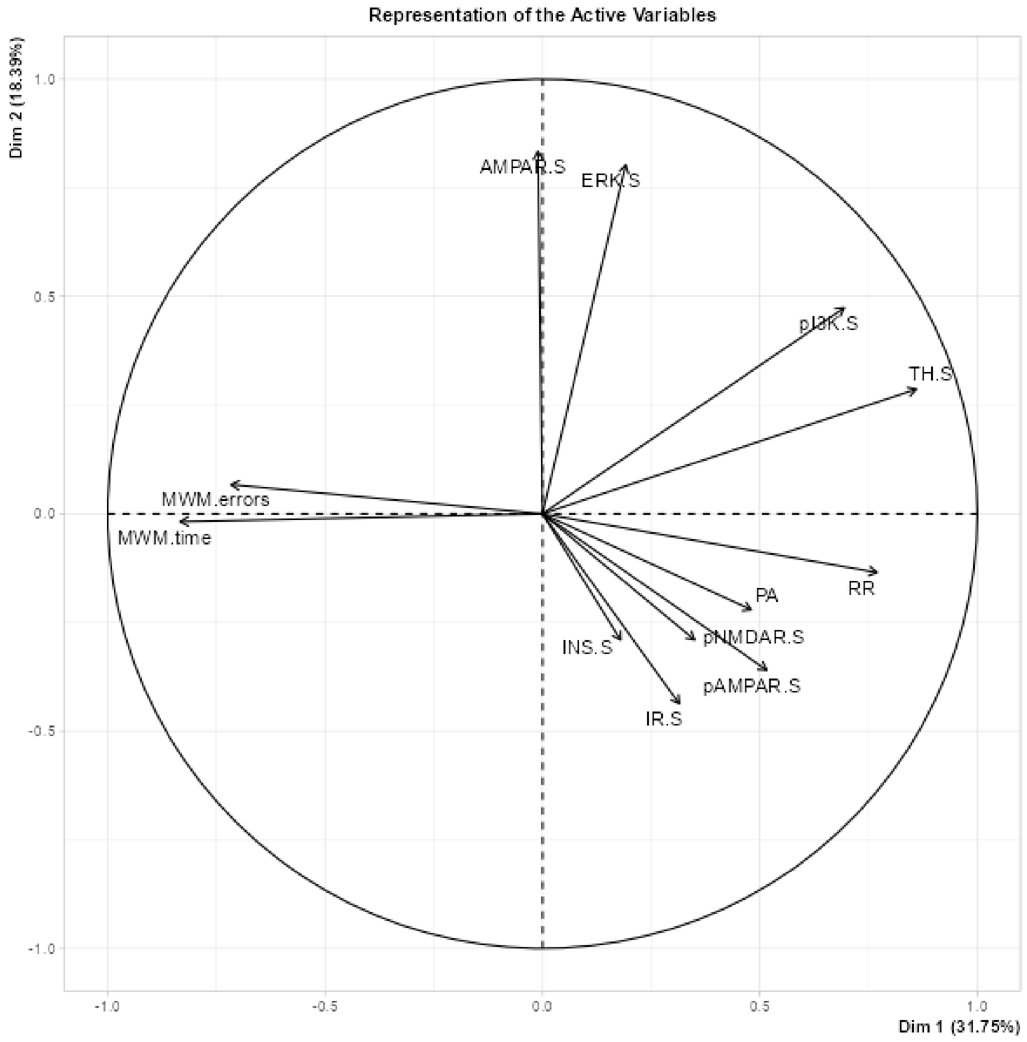


## Eigenvalue Decomposition

Eigenvalue and (Cumulative) Percentage of Variance

| | Eigenvalue | % of the variance | Cumulative % |
|---|---|---|---|
| Dim. 1 | 3.8104 | 31.754 | 31.8 |
| Dim. 2 | 2.2074 | 18.395 | 50.1 |
| Dim. 3 | 1.7699 | 14.749 | 64.9 |
| Dim. 4 | 1.1448 | 9.540 | 74.4 |
| Dim. 5 | 0.9675 | 8.063 | 82.5 |
| Dim. 6 | 0.5941 | 4.951 | 87.4 |
| Dim. 7 | 0.5712 | 4.760 | 92.2 |
| Dim. 8 | 0.3900 | 3.250 | 95.5 |
| Dim. 9 | 0.2781 | 2.318 | 97.8 |
| Dim. 10 | 0.1372 | 1.143 | 98.9 |
| Dim. 11 | 0.0879 | 0.732 | 99.7 |
| Dim. 12 | 0.0416 | 0.347 | 100.0 |

## Automatic Description of the Axes

```
$Dim.1
$quanti
           correlation   p.value
TH.S            0.8598 7.295e-08
RR              0.7702 1.073e-05
pI3K.S          0.6942 1.677e-04
pAMPAR.S        0.5161 9.826e-03
PA              0.4807 1.743e-02
MWM.errors     -0.7163 8.247e-05
MWM.time       -0.8334 4.286e-07

$quali
```

```
          R2   p.value
Group 0.8674 6.122e-10

$category
            Estimate   p.value
Group=CTRis    2.184 3.292e-05
Group=OHDA    -2.510 3.849e-07

attr(,"class")
[1] "condes" "list"

$Dim.2
$quanti
        correlation   p.value
AMPAR.S      0.8338 4.188e-07
ERK.S        0.8028 2.345e-06
pI3K.S       0.4735 1.942e-02
IR.S        -0.4370 3.275e-02

attr(,"class")
[1] "condes" "list"

$Dim.3
$quanti
         correlation   p.value
pNMDAR.S      0.7524 2.228e-05
IR.S          0.6203 1.223e-03
pAMPAR.S     -0.5868 2.576e-03

$quali
          R2  p.value
Group 0.3767 0.006986

$category
             Estimate  p.value
Group=OHDA     0.8445 0.045265
Group=CTRint  -1.1207 0.002355

attr(,"class")
[1] "condes" "list"

$Dim.4
$quanti
      correlation   p.value
PA         0.6011 0.0018934
INS.S     -0.6885 0.0001995

attr(,"class")
[1] "condes" "list"

$Dim.5
$quanti
           correlation  p.value
MWM.errors      0.5271 0.008133
PA              0.5012 0.012599
INS.S           0.4436 0.029919

attr(,"class")
[1] "condes" "list"
```

## Variable Tables

Contributions Table

| | Dim.1 | Dim.2 | Dim.3 | Dim.4 | Dim.5 |
|---|---|---|---|---|---|
| INS.S | 0.84887 | 3.8275 | 5.189 | 41.4115 | 20.33681 |
| TH.S | 19.40167 | 3.7200 | 1.650 | 0.6994 | 0.83426 |
| IR.S | 2.59693 | 8.6507 | 21.739 | 0.6636 | 0.53173 |
| ERK.S | 0.96298 | 29.2001 | 5.902 | 1.9352 | 0.00178 |
| pI3K.S | 12.64850 | 10.1589 | 2.144 | 0.0173 | 3.56381 |
| pAMPAR.S | 6.99123 | 5.8733 | 19.456 | 0.0197 | 6.56188 |
| AMPAR.S | 0.00270 | 31.4927 | 1.449 | 1.9262 | 10.05716 |
| pNMDAR.S | 3.22209 | 3.8184 | 31.984 | 8.5691 | 0.05113 |
| MWM.time | 18.22699 | 0.0148 | 0.475 | 3.1486 | 3.35491 |
| MWM.errors | 13.46675 | 0.2030 | 2.304 | 0.9579 | 28.71444 |
| PA | 6.06328 | 2.2149 | 2.450 | 31.5622 | 25.96301 |
| RR | 15.56801 | 0.8258 | 5.257 | 9.0893 | 0.02909 |

Correlation Matrix

| | | MWM/time | MWM/errors | PA | RR | INS-HPC | TH-HPC | IR-HPC | ERK-HPC | pI3K-HPC | pAMPAR-HPC | AMPAR-HPC | pNMDAR-HPC |
|---|---|---|---|---|---|---|---|---|---|---|---|---|---|
| MWM/time | Spearman's rho | — | | | | | | | | | | | |
| | p-value | — | | | | | | | | | | | |
| MWM/errors | Spearman's rho | 0.533 ** | — | | | | | | | | | | |
| | p-value | 0.007 | — | | | | | | | | | | |
| PA | Spearman's rho | -0.132 | -0.226 | — | | | | | | | | | |
| | p-value | 0.539 | 0.287 | — | | | | | | | | | |
| RR | Spearman's rho | -0.558 ** | -0.288 | 0.065 | — | | | | | | | | |
| | p-value | 0.005 | 0.172 | 0.763 | — | | | | | | | | |
| INS-HPC | Spearman's rho | 0.272 | 0.057 | -0.130 | -0.375 | — | | | | | | | |
| | p-value | 0.198 | 0.792 | 0.544 | 0.071 | — | | | | | | | |
| TH-HPC | Spearman's rho | -0.161 | -0.297 | 0.192 | 0.652 *** | -0.234 | — | | | | | | |
| | p-value | 0.453 | 0.159 | 0.369 | < .001 | 0.270 | — | | | | | | |
| IR-HPC | Spearman's rho | 0.049 | -0.316 | 0.364 | 0.211 | 0.217 | 0.243 | — | | | | | |
| | p-value | 0.820 | 0.132 | 0.081 | 0.323 | 0.308 | 0.252 | — | | | | | |
| ERK-HPC | Spearman's rho | 0.321 | 0.048 | -0.088 | -0.383 | 0.056 | -0.349 | 0.155 | — | | | | |
| | p-value | 0.126 | 0.824 | 0.681 | 0.065 | 0.796 | 0.095 | 0.468 | — | | | | |
| pI3K-HPC | Spearman's rho | -0.280 | -0.024 | 0.074 | 0.289 | 0.205 | 0.017 | 0.418 * | -0.259 | — | | | |
| | p-value | 0.185 | 0.911 | 0.731 | 0.170 | 0.334 | 0.937 | 0.043 | 0.221 | — | | | |
| pAMPAR-HPC | Spearman's rho | -0.402 | -0.362 | -0.071 | 0.458 * | 0.033 | 0.417 * | -0.173 | -0.312 | -0.023 | — | | |
| | p-value | 0.052 | 0.082 | 0.743 | 0.024 | 0.879 | 0.044 | 0.417 | 0.138 | 0.914 | — | | |
| AMPAR-HPC | Spearman's rho | -0.016 | -0.135 | 0.183 | -0.135 | -0.104 | -0.217 | 0.215 | 0.378 | -0.002 | -0.412 * | — | |
| | p-value | 0.942 | 0.528 | 0.392 | 0.528 | 0.626 | 0.308 | 0.312 | 0.069 | 0.995 | 0.046 | — | |
| pNMDAR-HPC | Spearman's rho | -0.477 * | -0.348 | 0.343 | 0.217 | 0.041 | 0.033 | 0.614 ** | -0.188 | 0.583 ** | -0.180 | 0.188 | — |
| | p-value | 0.033 | 0.133 | 0.138 | 0.357 | 0.866 | 0.891 | 0.005 | 0.426 | 0.008 | 0.445 | 0.426 | — |

*Note.* * $p < .05$, ** $p < .01$, *** $p < .001$

## Correlation Matrix

Correlation Matrix

| | | MWM/time | MWM/errors | PA | RR | INS-HPT | TH-HPT | IR-HPT | ERK-HPT | pI3K-HPT | pAMPAR-HPT | AMPAR-HPT | pNMDAR-HPT |
|---|---|---|---|---|---|---|---|---|---|---|---|---|---|
| MWM/time | Spearman's rho | — | | | | | | | | | | | |
| | p-value | — | | | | | | | | | | | |
| MWM/errors | Spearman's rho | 0.533 ** | — | | | | | | | | | | |
| | p-value | 0.007 | — | | | | | | | | | | |
| PA | Spearman's rho | -0.132 | -0.226 | — | | | | | | | | | |
| | p-value | 0.539 | 0.287 | — | | | | | | | | | |
| RR | Spearman's rho | -0.558 ** | -0.288 | 0.065 | — | | | | | | | | |
| | p-value | 0.005 | 0.172 | 0.763 | — | | | | | | | | |
| INS-HPT | Spearman's rho | 0.238 | -0.056 | -0.003 | 0.011 | — | | | | | | | |
| | p-value | 0.264 | 0.795 | 0.990 | 0.959 | — | | | | | | | |
| TH-HPT | Spearman's rho | -0.450 * | -0.498 * | 0.150 | 0.328 | 0.045 | — | | | | | | |
| | p-value | 0.027 | 0.013 | 0.484 | 0.118 | 0.834 | — | | | | | | |
| IR-HPT | Spearman's rho | -0.258 | 0.003 | 0.040 | 0.167 | 0.179 | 0.265 | — | | | | | |
| | p-value | 0.224 | 0.990 | 0.852 | 0.434 | 0.401 | 0.210 | — | | | | | |
| ERK-HPT | Spearman's rho | -0.362 | -0.146 | -0.292 | 0.207 | 0.103 | 0.444 * | 0.388 | — | | | | |
| | p-value | 0.083 | 0.495 | 0.166 | 0.331 | 0.629 | 0.031 | 0.062 | — | | | | |
| pI3K-HPT | Spearman's rho | 0.045 | -0.021 | -0.047 | -0.084 | 0.477 * | 0.210 | 0.414 * | 0.255 | — | | | |
| | p-value | 0.833 | 0.923 | 0.826 | 0.695 | 0.020 | 0.322 | 0.045 | 0.229 | — | | | |
| pAMPAR-HPT | Spearman's rho | 0.023 | 0.299 | -0.062 | -0.144 | -0.359 | 0.127 | 0.177 | -0.143 | -0.056 | — | | |
| | p-value | 0.916 | 0.156 | 0.775 | 0.503 | 0.085 | 0.553 | 0.405 | 0.505 | 0.796 | — | | |
| AMPAR-HPT | Spearman's rho | 0.467 * | 0.303 | -0.495 * | -0.190 | 0.157 | -0.322 | -0.037 | -0.071 | -0.027 | -0.045 | — | |
| | p-value | 0.021 | 0.150 | 0.014 | 0.374 | 0.463 | 0.125 | 0.863 | 0.740 | 0.901 | 0.834 | — | |
| pNMDAR-HPT | Spearman's rho | -0.315 | -0.063 | 0.049 | 0.181 | -0.030 | 0.130 | 0.709 *** | 0.171 | 0.462 * | 0.413 * | -0.036 | — |
| | p-value | 0.133 | 0.769 | 0.820 | 0.398 | 0.888 | 0.542 | < .001 | 0.422 | 0.024 | 0.046 | 0.869 | — |

*Note.* * p < .05, ** p < .01, *** p < .001

## Correlation Matrix

Correlation Matrix

| | | MWM/time | MWM/errors | PA | RR | INS-S | TH-S | IR-S | ERK-S | pI3K-S | pAMPAR-S | AMPAR-S | pNMDAR-S |
|---|---|---|---|---|---|---|---|---|---|---|---|---|---|
| MWM/time | Spearman's rho | — | | | | | | | | | | | |
| | p-value | — | | | | | | | | | | | |
| MWM/errors | Spearman's rho | 0.533 ** | — | | | | | | | | | | |
| | p-value | 0.007 | — | | | | | | | | | | |
| PA | Spearman's rho | -0.132 | -0.226 | — | | | | | | | | | |
| | p-value | 0.539 | 0.287 | — | | | | | | | | | |
| RR | Spearman's rho | -0.558 ** | -0.288 | 0.065 | — | | | | | | | | |
| | p-value | 0.005 | 0.172 | 0.763 | — | | | | | | | | |
| INS-S | Spearman's rho | -0.213 | -0.164 | 0.072 | 0.409 * | — | | | | | | | |
| | p-value | 0.318 | 0.443 | 0.738 | 0.047 | — | | | | | | | |
| TH-S | Spearman's rho | -0.519 ** | -0.601 ** | 0.339 | 0.584 ** | 0.404 | — | | | | | | |
| | p-value | 0.009 | 0.002 | 0.106 | 0.003 | 0.051 | — | | | | | | |
| IR-S | Spearman's rho | -0.221 | -0.010 | 0.157 | 0.069 | 0.471 * | 0.106 | — | | | | | |
| | p-value | 0.299 | 0.964 | 0.463 | 0.749 | 0.020 | 0.621 | — | | | | | |
| ERK-S | Spearman's rho | -0.184 | -0.306 | -0.150 | 0.193 | 0.113 | 0.563 ** | -0.065 | — | | | | |
| | p-value | 0.389 | 0.147 | 0.484 | 0.367 | 0.600 | 0.005 | 0.762 | — | | | | |
| pI3K-S | Spearman's rho | -0.610 ** | -0.379 | 0.222 | 0.309 | 0.224 | 0.594 ** | 0.121 | 0.411 * | — | | | |
| | p-value | 0.002 | 0.068 | 0.296 | 0.142 | 0.293 | 0.003 | 0.572 | 0.047 | — | | | |
| pAMPAR-S | Spearman's rho | -0.368 | -0.455 | 0.582 ** | 0.579 ** | 0.386 | 0.200 | 0.072 | -0.172 | 0.054 | — | | |
| | p-value | 0.121 | 0.050 | 0.009 | 0.009 | 0.104 | 0.410 | 0.770 | 0.480 | 0.826 | — | | |
| AMPAR-S | Spearman's rho | -0.050 | -0.063 | 0.022 | -0.024 | -0.162 | 0.203 | -0.335 | 0.477 * | 0.302 | 0.042 | — | |
| | p-value | 0.817 | 0.770 | 0.918 | 0.912 | 0.449 | 0.341 | 0.110 | 0.020 | 0.152 | 0.865 | — | |
| pNMDAR-S | Spearman's rho | -0.079 | -0.125 | 0.313 | -0.066 | 0.181 | 0.219 | 0.651 ** | 0.093 | 0.162 | -0.059 | -0.386 | — |
| | p-value | 0.719 | 0.570 | 0.146 | 0.766 | 0.408 | 0.313 | 0.001 | 0.672 | 0.458 | 0.818 | 0.070 | — |

*Note.* * $p < .05$, ** $p < .01$, *** $p < .001$
